# Characteristics of built food environments associated with alternative protein food choices: a systematic review

**DOI:** 10.1186/s12966-024-01606-6

**Published:** 2024-05-16

**Authors:** Hanna Zaleskiewicz, Ewa Kulis, Maria Siwa, Zofia Szczuka, Anna Banik, Francesca Grossi, Polymeros Chrysochou, Bjørn Tore Nystrand, Toula Perrea, Antonella Samoggia, Arlind Xhelili, Athanasios Krystallis, Aleksandra Luszczynska

**Affiliations:** 1 Faculty of Psychology CARE-BEH Center for Applied Research On Health Behavior and Health, SWPS University, 30B Ostrowskiego Street, Wroclaw, 53238 Poland; 2 Collaborating Centre On Sustainable Consumption and Production (CSCP), 30 Hagenauer Street, Wuppertal, E42107 Germany; 3 Department of Management, MAPP Centre, Aarhus University, 4 Fuglesangs Allé, Aarhus V, DK8210 Denmark; 4 American College of Greece Research Center, 6 Gravias Street, GR15342 Aghia Paraskevi, Athens, Greece; 5 Møreforsking, P.O. Box 5075, Ålesund, NO6021 Norway; 6 Department of Agriculture and Food Sciences, University of Bologna, 33 Via Zamboni, Bologna, T40126 Italy

**Keywords:** Alternative protein food, Built environment, Systematic review, Nutrition behavior

## Abstract

**Background:**

This systematic review contributes to the understanding of the characteristics of built food environments that may be associated with choices of alternative protein foods (APF). Using the built food environment typology proposed by Downs et al., we investigated various environmental structures (e.g., supermarkets, other retailers, farmers’ markets, restaurants, schools, and online vendors) and the characteristics that may facilitate or hinder consumers’ choices. For example, facilitators and barriers may refer to the physical characteristics of environmental structures, food presentation practices, the organizational strategies or policies operating in the setting, or the actions that retailers or consumers engage in while selling, serving, choosing, trying, or purchasing APF in these environmental structures.

**Methods:**

A systematic review (PROSPERO database preregistration; no. CRD42023388700) was conducted by searching 13 databases for peer-reviewed journals focusing on the fields of economics and business, agriculture, medical sciences, and social sciences. Data searches, coding, and quality evaluations were conducted by at least 2 researchers. A total of 31 papers (36 original studies) were included. The risk of bias was evaluated with the Joanna Briggs Institute quality evaluation tool, with 24 publications presenting low risk of bias.

**Results:**

The findings indicate that perceived and actual availability facilitate consumers’ APF choices across a built food environment. Several barriers/facilitators were associated with APF choices in specific types of built food environments: the way food is presented in produce sections (supermarkets), consumer habits in terms of green and specialty shopping (grocery stores), and mismatches among retailer actions in regard to making APF available in one type of food environment structure (e-commerce) and consumers’ preferences for APF being available in other food environment structures (supermarkets, grocery stores). The effect of a barrier/facilitator may depend on the APF type; for example, social norms regarding masculinity were a barrier affecting plant-based APF choices in restaurants, but these norms were not a barrier affecting the choice of insect-based APF in restaurants.

**Conclusions:**

Addressing barriers/facilitators identified in this review will help in developing environment-matching interventions that aim to make alternative proteins mainstream.

**Trial registration:**

PROSPERO database registration: #CRD42023388700.

**Supplementary Information:**

The online version contains supplementary material available at 10.1186/s12966-024-01606-6.

## Background

As highlighted by the EAT-Lancet Commission Report [1], the role of food in shaping human health and environmental sustainability is pivotal. The dietary shift that is key to securing healthier societies and more sustainable food production can be achieved by reducing the consumption of animal-based proteins (such as meat) and incorporating alternative protein sources (e.g., plant-based) into the daily diet [1]. For example, the EAT-Lancet Commission Report [1] suggested that transitioning from a diet where meat constitutes the main protein source to a diet with plant-based proteins would substantially reduce any-cause mortality risks, as well as the risk of stroke, type 2 diabetes, and cardiovascular diseases. Considering greenhouse emissions, land use, energy use, and acidification potential, soybeans, legumes, grains, and vegetables rich in proteins have the lowest environmental effects per serving, whereas meat from ruminant livestock has the greatest environmental effect [1]. Alternative protein foods (APF) encompass a wide range of protein concentrates derived from various sources, such as insects, krill, microbial biomass, mushrooms, fungi, and plants such as peas or rapeseed [2]. The term “alternative protein” often excludes cultured meat due to the ongoing debate about the environmental benefits of its production [2].

The concept of the “food environment” is usually applied to investigate linkages among built environment, social, and political contexts and dietary choices and their consequences for human health and the natural environment [3–6]. The physical built environment has a prominent role in the overall food environment [5, 6], and its characteristics constitute a setting where the social and political contexts operate together [7]. The various taxonomies explaining the physical food environment exhibit some similarities. For example, McKinnon et al. [6] categorized physical food environments into the following types: food stores, restaurants, schools, and worksite environments. A taxonomy proposed by Downs et al. [5] considers formal structures, such as supermarkets, hypermarkets, other retailers, farmers’ markets, restaurants, institutional and public procurement settings, mobile vendors, and online vendors, as well as informal environmental structures such as wet markets.

Between 2013 and 2022, major retailers (e.g., Walmart, Carrefour, and Tesco) started selling their own plant-based alternative protein food ranges, whereas major fast food companies (e.g., Yum!, the owner of KFC and Pizza Hut) have established commercial relationships with plant-based food producers and launched plant-based protein products [3]. In 2022, Burger King opened its first plant-only outlet in London, England [3]. Thus, APF is becoming more readily available for consumers in various built food environmental structures.

In addition to identifying where specific dietary choices occur (e.g., [5]), taxonomies of the built food environment should be complemented by the evidence-based characteristics of the respective structures that may facilitate or hinder dietary shifts toward specific choices (e.g., an increase in APF intake). The latter approach is in line with the context and implementation of the complex intervention approach [7], which highlights the role of the setting (a structure in a physical environment) and its contextual characteristics in codetermining the efficacy of any actions promoting new nutrition behaviors. The built food environment taxonomy proposed by Downs et al. [5] assumes that the characteristics of the food environment may include general features of built environment structures (e.g., availability, affordability, and sustainability of food products). Other approaches indicate that the characteristics of the “setting” (or the structure of the built food environment) may be divided into macro- (e.g., national policies addressing respective settings), meso- (e.g., ale strategies adopted by a retail chain), and microlevels (individual) [7]. Characteristics at the individual level may refer to the attributes of various food system actors (e.g., producers, retailers, and consumers).

Existing systematic reviews have explored the associations between behaviors such as increased intake of plant-based proteins or meat reduction and food promotion strategies (e.g., the recipe design, product labeling, or sensory characteristics of APF). For example, a review of 18 intervention studies investigated the microenvironmental characteristics associated with reducing meat consumption [8]. Strategies such as reducing meat portion sizes, altering the sensory characteristics of meat and meat alternatives, and providing meat-free options were associated with a reduction in meat intake. Notably, making meat alternatives available has been found to have a sustained effect [8]. Similarly, Stiles et al. [9] reviewed the effectiveness of strategies aimed at decreasing animal protein food and increasing plant protein food in food service settings, including menu redesign (increasing availability of nonmeat proteins on the menu), recipe redesign, service redesign (to improve sustainability), menu labeling (e.g., “a climate choice”), and prompts at the point of sale (e.g., “dish of the day”). The menu offer redesign strategy demonstrated the most substantial and significant effects [9].

Findings from previous reviews [8, 9] indicated that increased availability of APF facilitated changes in protein intake (meat reduction and increased plant protein intake). However, these reviews do not provide insights into whether these effects apply across different built environments (e.g., school canteens, restaurants, or supermarkets) or whether certain strategies are more impactful in specific settings (e.g., restaurants vs. supermarkets). Furthermore, these reviews do not delve into the barriers that may hinder APF uptake, and it remains unclear whether these findings can be generalized to other APF products, such as insect-based APF.

The intake and acceptability of insect-based APF in developed countries differ from what has been observed for African, Latin American, and Southeast Asian countries [10]. Consumers in the latter countries collect insects from uncultivated wild areas and sell them via informal markets [5]. These practices contrast with the strictly regulated formal market of insect-based APF in Western countries [11]. Due to these differences in the physical food environments where APF choices occur, our review focuses on built food environments in Western countries and developed Asian countries with strictly regulated formal APF markets (i.e., Japan).

The literature lacks a synthesis of evidence of characteristics that may be linked to an increase in the uptake of APF in specific settings. It remains unclear whether these characteristics are common or specific for some physical environmental structures and whether they are generalizable across different types of alternative proteins (e.g., plant-based vs. insect-based APF). A synthesis of evidence aids in the development of interventions promoting APF by identifying evidence-based characteristics that may facilitate or hinder APF consumption.

Using the systematic review method, the aim of this study was to identify the characteristics of the structures of the built food environment that may act as either barriers or facilitators for choosing APF products. In particular, we investigated (a) informal market food environments (wet markets, street vendors, kiosks, and mobile vendors) and (b) formal market food environments (supermarkets, hypermarkets, retailers, farmers’ markets, restaurants, institutional public procurement settings, mobile vendors, and online vendors) (cf. [5]). Furthermore, we sought to explore any built food environment characteristics that have been tested for their associations with consumers’ APF choices.

## Methods

### Materials and general procedures

This study was conducted following the guidelines of the Preferred Reporting Items for Systematic Reviews and Meta-Analyses (PRISMA) [12]. The findings presented are part of a broader systematic review, which was registered with the PROSPERO database under the registration number #CRD42023388700. The overarching goal of this systematic review is to identify the physical environmental characteristics related to consumers’ APF choices.

### Search strategy

We conducted a systematic search of 11 databases containing peer-reviewed journals from the fields of economics and business, agriculture, medical sciences, and social sciences (Academic Search Ultimate, PsycInfo, PsycArticles Business Source Ultimate, Agricola, GreenFILE, Health Source: Nursing Academic Edition, SocINDEX, MEDLINE, MasterFILE Premier, and Academic Research Source eJournals), which were accessed through the EBSCO platform. Separate searches of 2 additional databases, Web of Science and SCOPUS, were conducted following the primary search. Our search included documents and articles published up to March 2023.

The search strategy adopted three groups of keywords: (1) *alternative protein food* (e.g., "seaweed*" OR "alga*" OR "insect*" OR "lupin*" OR lentil* OR "mealworm”), (2) *physical built environment* (e.g., "shop*" OR "retail*" OR "cater*" OR "restaurant*" OR "supermarket") and (3) *consumer or behavior-related* (e.g., "intake" OR "food" OR "consume*" OR "eat" OR "sale") keywords. For the full list of keywords, see Additional File 1. These keywords were selected using existing reviews on APF [13–15] and the food environment typology by Downs et al. [5]. Researchers from the fields of consumer sciences, food sciences, and nutrition from the LIKE-A-PRO consortium were consulted regarding the appropriateness of the keywords [16].

The feasibility of the search string was pretested across the databases before the search was initiated. The aim of this approach was to capture a wide range of relevant articles across the databases. This approach increased the number of identified entries and, thus, minimized the likelihood of excluding relevant documents during the initial screening stages.

To ensure the robustness of the search, we performed manual searches of references within the full texts of original studies assessed for inclusion, as well as complementary nonsystematic searches of Google Scholar using the same keywords as used in the databases. Finally, we searched the CORDIS and Open Research Europe (ORE) databases for open peer-reviewed articles. The keywords were modified to fit the character limits (up to 50 characters in length) imposed by CORDIS and ORE.

### Inclusion and exclusion criteria

The following inclusion criteria were applied: (1) peer-reviewed English-language original quantitative or qualitative studies; (2) studies addressing alternative protein-based foods, including proteins that are land or sea plant-based, fungi-based, bacteria-based, or based on any other alternative protein sources, such as krill, as well as combinations of meat- and plant-based proteins; and (3) studies investigating built and/or physical environmental structures, based on the built food environment typology by Downs et al. [5], where European consumers made choices regarding APF. Original studies were included if (4) they discussed any type of link between the characteristics of the food environment and any (a) indicators of consumers’ choices, such as perceived display/ways of exhibiting food in the built environment, intention to buy, intention to eat, actual intake, and actual sales, or (b) indicators of food availability in the respective food environment.

The exclusion criteria were as follows: (1) studies that did not report any original data, such as reviews or position papers; (2) dissertations, protocols, conference materials, or book chapters; (3) studies focusing solely on reducing meat intake without investigating how proteins will be supplemented in the diet by APF products; (4) studies focusing on increasing the intake of fruits or vegetables without specific data on plant-based protein sources; (5) studies solely addressing the physical environment in Asia, Africa, or South America, entailing locally collected wild-living insects and their local consumption or local retail; (6) studies involving novel foods without mentioning that the food is made of/with alternative protein sources, e.g., novel drinks based on sea buckthorn; (7) studies addressing consumers’ choices regarding laboratory-based, in vitro-grown meat, with no alternative proteins added; (8) studies focusing on geographical factors, such as between-country or between-region differences; and (9) studies investigating APF as supplements or animal feed.

### Data collection and extraction

**Fig. 1:**
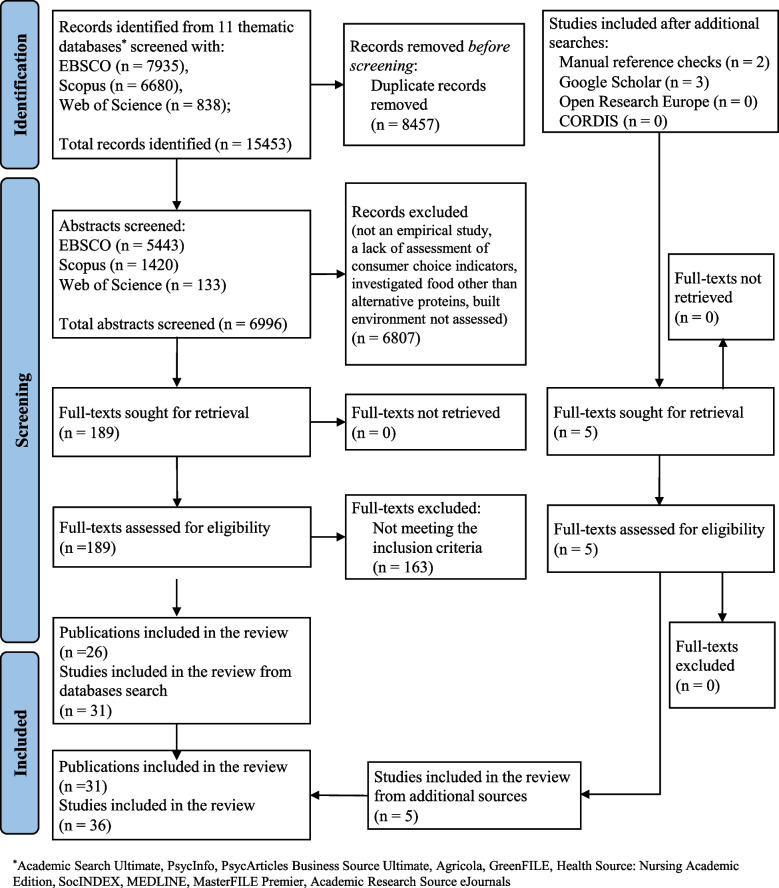
PRISMA flow diagram of the article search and selection process for the systematic review

Figure 1 illustrates the data selection process. The databases were independently searched by three researchers (HZ, EK, MS), and the searches were unsystematically checked by a fourth researcher (AL). The initial search yielded *k* = 7,935 records obtained from searches of 11 databases using the EBSCO search engine, *k* = 838 from the Web of Science, and *k* = 6,680 from SCOPUS. All abstracts were screened by two researchers (randomly assigned from a group of five researchers, HZ, EK, ZS, MS, and AB) to identify potentially relevant studies. Any conflicts regarding the inclusion of a document were resolved through discussions with a fourth researcher (AL). Next, three researchers (AL and two randomly assigned researchers from a group of five researchers—HZ, EK, ZS, MS, and AB—independently read the full-text versions of the articles and determined their match with the inclusion criteria. Additional searches for original peer-reviewed studies were conducted through screening the references of articles evaluated for inclusion independently by PC and TP and searching Google Scholar, CORDIS, and ORE databases (conducted by AL or HZ).

Thirty-one publications reporting 36 independent studies were included (Fig. 1). Two articles [17, 18*] reported findings from the same study; only the latest publication [18*] was included. We included two original studies reported in a publication by Vandenbroele et al. [19*], three original studies reported by Motoki et al. [20*], and three original studies reported by Baker et al. [21*].

The following data were extracted to address the study’s objectives: study population characteristics, country of data collection, design of the original study and type of methods used to collect data, the period when the data were collected, the type of alternative proteins investigated, the type of built environment and its characteristics, indicators of the consumers’ choices, and key results (see Supplementary Table 1, Additional File 1). Data extraction was conducted by two researchers (HZ and AL). Any disagreements during these stages were resolved by a consensus method (searching for possible rating errors, followed by discussion and arbitration by a third researcher, AB) [22].

### Data coding, analysis, and synthesis

The data retrieved from each original study were coded according to four categories: (1) a type of alternative protein food products; (2) a type of built food environmental structure; (3) a characteristic of the built food environment that was related to consumers’ choices of alternative proteins; and (4) a type of the consumers' choice indicator. Additional File 1 presents the definitions, theoretical background, and categories applied during the coding procedures.

The included material was heterogeneous for each type of built environmental structure (e.g., supermarkets) in terms of consumers’ choice indicators, types of alternative proteins, and built environment characteristics. Therefore, a meta-analysis was not feasible. We employed narrative synthesis methods instead [23, 24] (for details, see Additional File 1).

### Risk of bias and quality assessment

The methodological quality and risk of bias in the included studies were assessed using the Joanna Briggs Institute Critical Appraisal Tools [25] for both cross-sectional and qualitative studies. These tools are appropriate for evaluating qualitative and quantitative cross-sectional studies (no observational longitudinal studies were included, *k* = 6 manuscripts reported experimental studies). Each study was evaluated based on eight criteria and overall quality (good, fair, or poor). For details, see Additional File 1.

The methodological quality (risk of bias) of the included publications and respective studies was assessed by two pairs of independent reviewers (PC and TP or AB and MS). Studies were scored according to the critical appraisal questions. Disagreements were resolved by discussion or by consulting a third researcher (AL). The overall risk of bias for the included studies was determined using the following cutoffs: low risk of bias, if at least 70% of the assessed criteria were met; moderate risk, if 50–69% of the criteria were met; and high risk, if less than 50% of the criteria were met.

## Results

### Description of included studies

A total of *k* = 36 original studies were included. Additional File 1 presents the general descriptive information about the included studies and the results of coding referring to consumer choice indicators (e.g., acceptability, intention to eat, intention to buy, etc.). The enrolled populations were heterogeneous, with a total of *N* = 113,984, sample sizes ranging between 15 and < 100,000 (M = 3,453.09; SD = 17,344.40), and ages ranging from 7 to 90 years. Almost half of the studies were cross-sectional (*k* = 15, 41.6%), *k* = 11 (30.5%) were experimental, *k* = 9 (25%) were qualitative, and *k* = 2 (5.5%) were mixed method studies.

#### Types of APF addressed in the original studies

Across the original studies, *k* = 17 studies discussed plant-based alternative protein products, *k* = 15 studies addressed insect-based alternative protein products, *k* = 1 accounted for hybrid meat products (with meat and plant-based meat replacement combined), *k* = 4 analyzed foods from a combination of either plant-based proteins or insect-based sources, and *k* = 3 focused on a broader category of novel food, including either plant-based or insect-based APF or APF from various sources.

#### Built environmental structures studied in the included research

None of the studies addressed informal market structures (wet markets, street vendors, kiosks, or mobile vendors), whereas *k* = 31 (all studies) addressed the formal market food environment. In particular, *k* = 6 studies addressed supermarkets, *k* = 10 discussed other food retailer structures (e.g., grocery stores),* k* = 2 addressed farmers’ markets, *k* = 17 addressed restaurants, *k* = 2 addressed institutional and public procurement (schools) settings, *k* = 4 addressed online vendors, *k* = 1 addressed vending machines, and *k* = 4 addressed food festivals.

#### The risk of bias

The findings indicated that 24 of the included publications presented a low risk of bias, three studies had a moderate risk, and four studies had a high risk of bias (for details, see Additional File 2). An interrater reliability analysis between the independent reviewers’ scores was performed using Cohen’s kappa (values larger than 0.60 indicate strong agreement). The analysis showed strong agreement between the two raters, with κ = 0.69 (95 CI: [0.42, 0.96]). No study was excluded on the basis of the quality assessment.

### Findings for the formal market food environment

The extracted data were organized using the following grouping categories: (a) type of physical built environmental structure, using Downs et al.’s [5] taxonomy, and (b) type of organizational characteristics of the setting, i.e., meso-level (e.g., availability, product location, and promotion strategies used in the setting), as well as microlevel (e.g., stakeholders’ perceptions of availability, social norms, shopping habits, preferences for location, and occasion to eat APF) [7]. The findings for different types of APF (plant-based, insect-based, and other APF sources) are discussed separately to determine potential differences.

#### Supermarkets

The characteristics of supermarkets linked with consumers’ APF choices were reported in six studies [26,* 27,* 28,* 29,* 30,* 31*]. Three studies discussed plant-based alternative proteins, and three discussed insect-based proteins. All studies focused on the APF presentation in supermarkets, the period during which a product was available for sale, and the availability of the products.

##### Availability (plant-based APF)

The number of APF products available in supermarkets has been on the rise. For example, Australian data indicate a doubling of APF (130% increase) between 2014 and 2021, with a 150% increase in plant-based meat replacements. However, the availability of tofu products has decreased over time [26*]. Most APF products have been available in supermarkets for an average of two years [26,* 27*]. Sausage is the most popular plant-based meat replacement [27*].

##### Product location, APF promotion, and ease of finding APF (plant-based APF)

Plant-based meat alternatives are allocated a less shelf space than other protein products. Plant-based APF products are placed in less prominent sections of supermarkets and are perceived as “hidden” by consumers. They are also less likely to have sale, quality, or promotional signage compared to traditional meat products [28*]. Plant-based meat alternatives are often placed in produce (fruits/vegetables) sections or among high-end products [28*].

Plant-based meat alternatives are perceived as difficult to locate in stores, especially compared to meat products, which is also attributed to inconsistencies in their placement within and across retail chains [28*].

##### Retailers’ beliefs and practices (plant-based APF)

Supermarket retailers await clear demand signals before introducing new APF products [28*]. Retailers believe that including APF in dairy and meat sections will reduce supermarket profits, and placing APF-based meat substitutes away from meat sections may address the concerns of vegetarians and vegans [28*].

##### Shopping habits and preferred location for intake (plant-based APF)

Consumers who typically purchase protein products in upscale supermarket chains are willing to pay more for plant-based APF products [29*]. In contrast, those who usually shop in discount supermarkets were less willing to pay for APF products, possibly due to lower quality perceptions [29*].

##### Retailers focus on e-commerce and perceived availability in supermarkets (insect-based APF)

For consumers, the focus of retailers on selling via e-commerce instead of in supermarkets may be perceived as a barrier [30*]. To enhance consumer trust and confidence, the widespread distribution of insect-based APF products in supermarkets and other grocery stores is recommended [30*]. Finally, consumers report that the lack of availability of insect-based APF in supermarkets is a barrier to the intention to eat [31*].

#### Grocery stores/other types of shops selling food

The characteristics of grocery stores associated with consumers’ choices of alternative protein food were reported in 11 studies, reported in nine publications [18,* 19,* 21,* 27,* 32,* 33,* 34,* 35,* 36*]. Six studies discussed plant-based alternative proteins, while five discussed insect-based proteins. The research focused on the APF presentation at retail points, the perceived availability of products, the preference for the type of point of sale/retail, and consumer shopping practices, including “green” shopping, specialty food shopping, and shopping off campus (students).

##### Product location and promotion (plant-based APF)

The placement of APF on shelves with vegetarian food or in produce departments results in lower sales of APF, while higher sales occur when the APF is placed in the meat section [19*]. In addition, an increase in APF sales was also achieved when sandwiches made with plant-based APF were placed in the same refrigerator as sandwiches with meat (compared to a separate refrigerator) or in a refrigerator visible from the shop entrance (versus with its back to the entrance) [19*].

##### Perceived availability (plant-based APF)

The perceived availability of food in locations where food is usually purchased is related to satisfaction with plant-based proteins [32*]. Research revealed that 25% of respondents indicated “not available where I usually buy food” as a key barrier to trying new plant-based APF [33*]. Furthermore, consumers who intended to purchase such foods claimed that the availability in their usual food shopping places was low. Availability was a significant factor for those respondents who were likely to purchase new protein alternatives, but it was weakly associated with consumer choice indicators among undecided consumers [33*].

##### Shopping habits (plant-based APF)

The level of approval of plant-based APF was greater among customers who regularly shop in specialty food stores [18*]. Students who shopped for food in grocery stores located outside of university campuses were more likely to purchase plant-based APF than those who shopped on campuses [35*].

##### Product promotion (insect-based APF)

Research has indicated no major differences in the intention to purchase or expected fondness for/attractiveness among products with visible insects on the package vs. pictures of an insect-based powder and a Latin name [21*]. Notably, the levels of intention to buy insect-based APF were relatively low [21,* 34,* 36*].

##### Preferred location for availability and shopping habits (insect-based APF)

Consumers indicated their preference for the availability of insect-based APF across different food outlets, such as grocery stores, convenience stores, and petrol stations, and disagreed that such products should be available via e-commerce only or in specialty shops [27,* 36*]. Frequent “green shopping” in grocery stores was positively associated with a greater willingness to buy, fondness for, and willingness to pay for APF [34*].

#### Farmers’ markets

Only two studies have investigated the characteristics of farmers’ markets linked to consumers’ APF choices [26,* 27*].

##### Preferred location for APF purchases

Porretta et al. [27*] reported that older consumers were willing to buy insect-based APF if they were available from local producers selling their products in small markets. In contrast, Aerni et al. [26*] reported that points of sale at railway stations in large cities (e.g., Zurich) had greater sales of plant-based alternative food than did small farmers’ markets in the same cities.

#### Restaurants

Seventeen studies (reported in 14 publications) addressed restaurant characteristics in relation to consumers’ choice of APF [18,* 20,* 21,* 37,* 38,* 39,* 40,* 41,* 42,* 43,* 44,* 45,* 46,* 47*]. Six focused on plant-based APF, five focused on insect-based APF, and six discussed APF from various sources. The investigation addressed preferences for restaurants, such as APF environments, a prognosis of availability by experts, consumer social norm conformity, restaurant image creation and promotion, and meal presentation.

##### Experts’ perceptions of the popularity of plant-based and insect-based APF

Research investigating haute cuisine restaurant trends suggested that expert panels (selected on the basis of their entry on the official Michelin Guide website) predict that alternative proteins (insect- and plant-based) will become a strong trend in major European restaurants [47*]. These experts anticipate that plant- and insect-based proteins will be served to a greater extent than in vitro/cultivated meat. Additionally, the trend toward using locally sourced ingredients is expected to continue, with ingredients from distant regions playing a less important role [47*].

##### Experts’ actions to create a positive social image of plant-based APF

Creating a positive social image for restaurants is a strategy for increasing consumer interest. For example, renowned chefs have recognized the potential of using microalgae-based APF as an ingredient in their cuisines since there is a growing audience of consumers interested in food novelty who identify themselves with chefs’ discourses about sustainability, ethnicity, and authenticity [46*]. Such celebrated restaurants may also help popularize the use of plant-based APF in casual or mid-range restaurants, as well as home-based dining [46*].

##### Perceived availability of plant-based APF

Perceived availability in the restaurant was identified as a determinant of consumers’ decisions to purchase plant-based APF in these restaurants. For example, consumers’ perceptions that plant-based APF is easy to find on restaurant menus were associated with a greater willingness to pay for this type of food [45*].

##### Eating-out habits and preferred location, occasion, and company for consumption (plant-based APF)

A greater frequency of eating in restaurants was related to a greater willingness to pay for plant-based meat alternatives [37*]. Among women, a higher frequency of dining out/going to restaurants with friends and family was related to perceiving microalgae-based food as healthy, sustainable, and nutritious, whereas among men, it was related to unfavorable perceptions (e.g., limited healthiness or nutritional values of algae-based foods) [18*]. The findings regarding gender differences align with previous research indicating that higher masculinity is associated with lower acceptance of social eating situations (e.g., restaurants) involving the consumption of nonmeat products among men [40*]. Weinrich and Elshiewy [18*] also noted that the relationship between APF perceptions and the frequency of dining out/going to restaurants with friends and family may also depend on the country where the study was conducted.

Research highlights the relevance of social norms and the social context in the consumption of plant-based APF in vegetarian/vegan restaurants [40*]. Young meat-eating men were more likely to eat plant-based APF burgers in vegetarian restaurants when encountering specific social cues, such as the presence of other men queuing for a veggie burger or dining with a female romantic partner [40*]. Conversely, visiting such restaurants and portraying oneself as dining in a vegetarian restaurant on social media was seen as a threat to masculinity [40*]. In line with this, omnivores and flexitarians indicated low acceptance rates for eating plant-based APF while dining in restaurants or during business lunches with coworkers [44*].

Consumers report that eating plant-based APF in more casual, private situations may be perceived as more appropriate than consuming this type of food during more celebratory occasions [44*]. These eating occasions may take place at home or in pubs, bars, or restaurants. For omnivores and flexitarians, eating alone, with friends, or with family members on a weekday is perceived as the best (and equally appropriate) environment to eat plant-based meat alternatives [44*]. However, for omnivores and flexitarians, eating plant-based meat alternatives for a family Sunday meal or at a barbecue party received similar, low appropriateness ratings [44*]. These results echo similar findings obtained by [40*], who highlighted the influence of social norms on the purchase of plant-based meat alternatives in restaurants.

##### Eating-out habits and preferred location, occasion, and company for consumption (various types of APF)

Restaurants were indicated as the second most preferred place for consumers to try various types of novel food, including insect-based and plant-based APF (food festivals were the most preferred, and homes, cafés, pubs, and bars were less preferred locations) [20*].

##### Availability of locations serving insect-based APF

Insect-serving restaurants were perceived as relatively rare, with only 31% of consumers agreeing that some European gourmet restaurants incorporate edible insects into their food preparation [42*].

##### Experts creating a positive social image of restaurants (insect-based APF)

The use of APF can serve as a strategic promotional tool for restaurants. This strategy may include pushing boundaries with unusual ingredients, encouraging customers to try small portions of novel foods, and offering refunds if the meal is unsatisfactory [41*]. Applying such strategies conveys a greater sense of freedom of choice to customers [41*]. A positive image of edible insect restaurants was related to a stronger intention to eat insects [43*]. Furthermore, being an environmental advocate (the ability to convince others to act for environmental conservation) was related to a better image of insect-serving restaurants [43*].

Studies have shown that the presence of visible insects in restaurant-served meals leads to a lower intention to purchase, lower expected liking, and lower attractiveness of the food compared to food with invisible insects and vague descriptions of insect-based ingredients [21*]. Restaurants serving insects often employ strategies such as name ambiguity for meals containing insects and deliberate beautification during their presentation (e.g., garnishing to obscure ingredients and reduce neophobic tendencies) [41*]. The absence of visible insects in restaurant-served meals was related to low perceived risks [21*] and low anxiety while eating the respective types of foods, which resulted in stronger intentions to (re)visit restaurants serving insect-based APF [38*].

##### Preferred location and company for consumption (insect-based APF)

Restaurants are perceived as the preferred places to eat insect-based APF products. When asked about how they would eat food made with edible insects, the majority of consumers stated, “with an expert”, followed by “in a restaurant”, and “with someone who knows how to prepare it” [39*].

#### Schools

Two studies [48,* 49*] focused on the availability of plant-based APF and the impact of school workshops among children aged 7–14 years. Other institutions or public procurement environments were not investigated.

##### Availability of plant-based APF

The studies revealed that public schools did not offer APF for lunch, whereas private schools offered them less than once per week [48*].

##### Delivery of education interventions regarding insect-based APF

An approximately 20% increase in readiness to choose insect-based APF for lunch was observed among children after brief (45 min) workshops addressing the reasons for and context of eating insects, followed by insect-based APF tasting, being delivered at schools [49*].

#### Online vendors

Four studies discussed aspects associated with consumers’ choices of APF [27,* 36,* 50,* 51*], with all of them reporting on insect-based APF products.

##### Availability of insect-based APF

Insect-based APF sales predominantly occur through online retail channels, with European producers using e-commerce as a distribution channel five times more frequently than physical sale points [51*]. However, this strategy may have consumer trust and credibility limitations. To enhance consumer trust and credibility, these products may need to be sold in supermarkets and local groceries instead of focusing on e-commerce [50*].

##### Preferred location for the purchase of insect-based APF

When consumers were asked about their preference for the availability of insect-based APF across different food sale points, they generally disagreed with the idea that this type of food should be exclusively available through e-commerce [27,* 36*].

#### Food festivals

Two studies [20,* 45*] highlighted the significance of food events and food festivals as highly approved environments for plant- and insect-based APF.

##### Preferred location or occasion for purchasing/trying plant-based and insect-based APF

Regarding willingness to try plant-based meat alternatives, consumers indicated the highest willingness to try them at food festivals, followed by restaurants (lower at home, cafés, bars, and pubs) [20*]. Consumers who reported an opportunity to consume seaweed or algae-based food during a gastronomic event or a trip are more likely to consume plant (algae)-based APF than those who did not participate in such events [45*]. Potential consumers who were asked about their preferred location to try insect-based APF reported that they were most willing to try them during food festivals, whereas significantly lower levels were reported for trying them at home or in restaurants/cafés/bars/pubs [20*].

#### Food vending machines

Research focusing on a specific type of food (algae-based breadsticks) suggests that customers consider this type of food a snack rather than a meal substitute and should be sold from vending machines [52*].

##### Informal market environment: wet markets, mobile vendors, street vendors, kiosks, vending machines, and farmers’ markets

There is no evidence directly linking wet markets, street vendors, kiosks, mobile vendors, and consumers’ behaviors and intentions to buy/pay for *plant-based or insect-based APF*. It seems plausible that the European food market is mostly formalized due to national and European Union-level regulations referring to food safety, labeling, and quality, particularly those referring to novel foods (cf. the European Commission Implementing Regulation, 2018/456 of 19 March 2018 [53]).

### Summary of findings

**Fig. 2:**
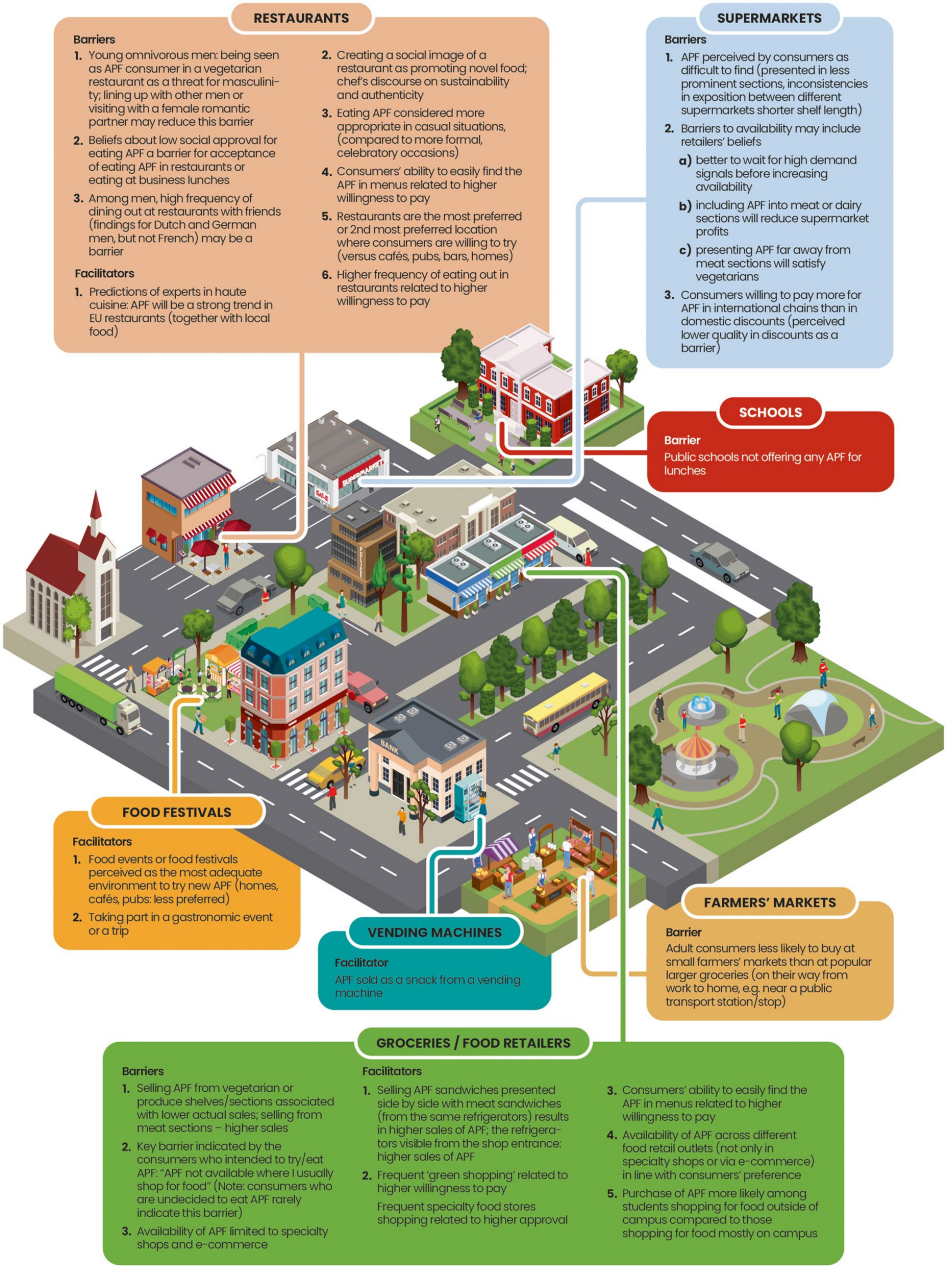
Built environment barriers to/facilitators of plant-based alternative protein foods (APF) consumer choices

**Fig. 3:**
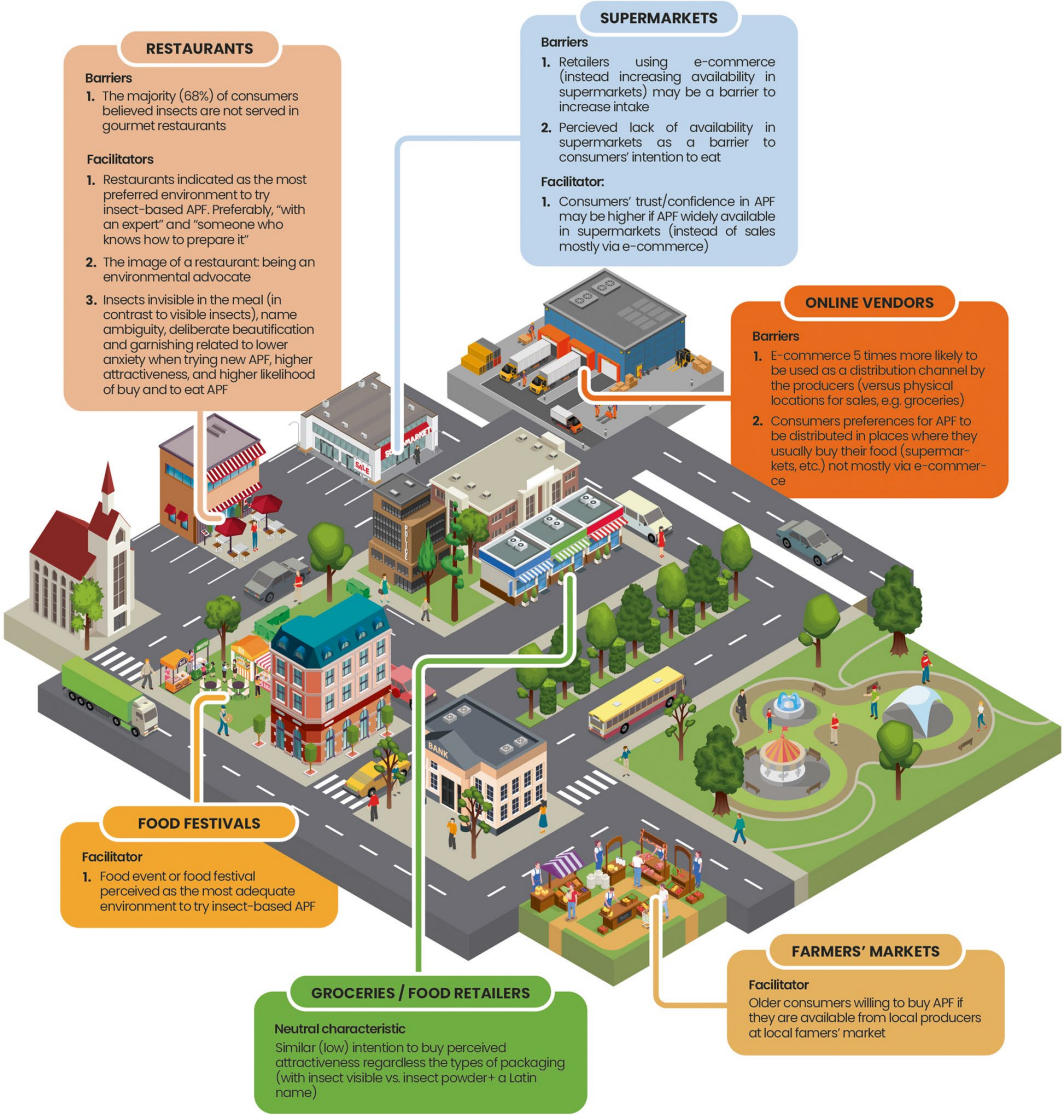
Build environment barriers to/facilitators of insect-based alternative protein foods (APF) consumer choices

Table 1 summarizes the findings, focusing on (a) the structures of the built food environment where consumers make APF choices and (b) barriers to and facilitators for choosing plant-based and insect-based APF, operating in the respective structures. Barriers and facilitators refer to: the physical characteristics of environmental structures; food presentation practices; organizational strategies or policies operating in the setting; and the actions and beliefs of retailers or consumers while selling, serving, choosing, trying, or purchasing APF in these environmental structures. The synthesis of the evidence indicated potential differences between the characteristics of the structures of the built environment that may be relevant for plant-based APF (Fig. 2) and those that may be relevant for insect-based APF (Fig. 3).

**Table 1:** Types of built food environments and factors associated with consumers’ APF choices

Type of built food environment	Type of alternative protein	The barriers and facilitators operating in the built food environment associated with consumers’ choices of the respective APF
**Supermarkets**	**Plant-based APF**	**Barriers:** 1. APF perceived by consumers as difficult to find (presented in less prominent sections, inconsistencies in exposition between different supermarkets, or shorter shelf length) 2. Barriers to availability may include retailers’ beliefs (a) better to wait for high demand signals before increasing availability (b) including APF in meat or dairy sections will reduce supermarket profits (c) presenting APF far away from meat sections will satisfy vegetarians 3. Consumers willing to pay more for APF in international chains than in domestic discount stores (perceived lower quality in discount stores as a barrier)
	**Insect-based APF**	**Barriers:** 1. Retailers using e-commerce (instead of increasing availability in supermarkets) may be a barrier to increased intake 2. Perceived lack of availability in supermarkets as a barrier to consumers’ intention to eat **Facilitators:** 1. Consumers’ trust/confidence in APF may be higher if APF are widely available in supermarkets (instead of sales mostly via e-commerce)
**Groceries/other food retailers**	**Plant- based APF**	**Barriers** 1. Selling APF from vegetarian or produce shelves/sections associated with lower actual sales; selling from meat sections – higher sales 2. Key barrier indicated by the consumers who intended to try/eat APF: “APF not available where I usually shop for food” (Note: consumers who are undecided to eat APF rarely indicate this barrier) 3. Availability of APF limited to specialty shops and e-commerce **Facilitators** 1. Selling APF products presented side by side with meat products (in the same refrigerators) results in higher sales of APF; the refrigerators visible from the shop entrance: higher sales of APF 2. Frequent ‘green shopping’ related to higher willingness to pay 3. Frequent specialty food store shopping related to higher approval 4. Availability of APF across different food retail outlets (not only in specialty shops or via e-commerce) in line with consumers’ preferences 5. Purchase of APF more likely among students shopping for food outside of campus compared to those shopping for food mostly on campus
	**Insect-based APF**	**Neutral characteristic** 1. Similar (low) intention to buy, perceived attractiveness regardless the types of packaging (with insect visible vs. insect powder + a Latin name)
**Farmer’s markets**	**Plant-based APF**	**Barrier** 1. Adult consumers are less likely to buy at small farmers’ markets than at popular larger grocery stores (e.g., on their way home from work, at/near the public transportation stop)
	**Insect-based APF**	**Facilitator** 1. Older consumers willing to buy APF if they are available from local producers at local famers’ markets
**Restaurants**	**Plant-based APF**	**Barriers** 1. Young omnivorous men: being seen as an APF consumer in a vegetarian restaurant as a threat for masculinity; lining up with other men or visiting with a female romantic partner may reduce this barrier 2. Beliefs about low social approval for eating APF is a barrier for acceptance of eating APF in restaurants or eating at business lunches 3. Among men, high frequency of dining out at restaurants with friends (findings for Dutch and German men, but not French) may be a barrier **Facilitators** 1. Predictions of experts in haute cuisine: APF will be a strong trend in EU restaurants (together with local food) 2. Creating a social image of a restaurant as promoting novel food; chef’s discourse on sustainability and authenticity 3. Eating APF considered more appropriate in casual situations, (compared to more formal, celebratory occasions) 4. Consumers’ ability to easily find the APF in menus related to higher willingness to pay 5. Restaurants are the most preferred or 2nd most preferred location where consumers are willing to try (versus cafés, pubs, bars, homes) 6. Higher frequency of eating out in restaurants related to higher willingness to pay
	**Insect-based APF**	**Barriers** 1. The majority (68%) of consumers believed insects are not served in gourmet restaurants **Facilitators** 1. Restaurants indicated as the most preferred environment to try insect-based APF. Preferably, “with an expert” and “someone who knows how to prepare it” 2. The image of a restaurant: being an environmental advocate 3. Insects invisible in the meal (in contrast to visible insects), name ambiguity, deliberate beautification and garnishing related to lower anxiety when trying new APF, higher attractiveness, and higher likelihood of buy and to eat APF
**Schools**	**Plant based APF**	**Barriers** 1. Public schools not offering any APF for lunches
**Online vendors**	**Insect-based APF**	**Barriers** 1. E-commerce 5 times more likely to be used as a distribution channel by the producers (versus physical locations for sales, e.g. groceries) 2. Consumers preferences for APF to be distributed in places where they usually buy their food (supermarkets, etc.) not mostly via e-commerce
**Food festivals**	**Plant-based APF**	**Facilitators** 1. Food events or food festivals perceived as the most adequate environment to try new APF (homes, cafés, pubs: less preferred) 2. Taking part in a gastronomic event or a trip
	**Insect based APF**	**Facilitators** 1. Food event or food festival perceived as the most adequate environment to try insect-based APF
**Vending machines**	**Plant-based APF**	**Facilitators** 1. APF sold as a snack from a vending machine

*Note*. *APF* alternative protein food

## Discussion

Complementing the proposal of Downs et al. [5] that lists built environment structures relevant for all food choices, the findings of this systematic review provide an overarching synthesis of the characteristics that can either promote or hinder consumers’ choices of APF products in restaurants, supermarkets, grocery stores, schools, farmers’ markets, or at food festivals (see Table 1). Consistent with existing typologies [5, 6, 54] and systematic reviews [8, 9], our study highlights that availability is the core characteristic of the built environment that facilitates consumers’ APF choices. Our review provides insights into how availability is shaped and how it influences the choices of different types of APF across different settings within the built food environment. Limited availability appears to constitute a common barrier observed across supermarkets, grocery stores, restaurants, and schools.

Our review indicates a mismatch between the APF supply and the APF demand that may be responsible for stagnated APF sales and low consumption. Retailers believe that increasing APF in supermarkets will harm profits [30*], and their subsequent decisions to sell APF via e-commerce [51*] result in low APF availability in groceries/supermarkets. Consumers, in turn, report low trust in products that are not available in grocery stores or supermarkets but are only available via e-commerce [50,* 55]. Consumers’ trust is a key determinant of buying and eating novel foods [56]. The limited availability of APF in locations where consumers usually shop for food results in low APF acceptance and low intention to buy or try APF [30,* 33,* 45*]. Consumers are less likely to choose an APF if the APF is perceived as difficult to find in supermarkets/grocery stores/on restaurant menus [30,* 33,* 45*]. The lack of actual availability of plant-based APF at schools [48*] may also result from the low trust of consumers in novel foods such as APF.

The current systematic review highlights that barriers and facilitators are specific for both the type of built environment and the type of APF (plant-based vs. insect-based). Regarding the type of structure in the built environment, our review indicates several specific social facilitators and barriers that operate in restaurants. In terms of facilitators, restaurants are preferred locations to try novel foods for the first time [39*], possibly because consumers perceive restaurants as places where experts prepare and serve food they can feel safe consuming [56]. The narrative created by chefs and the social image of restaurants promoting novel and sustainable foods [46*] may facilitate the use of APF in restaurants. Moreover, consumers’ beliefs about the importance of the sustainability of APF are among the key individual-level predictors of consumers’ choices of APF (cf., e.g., [57]). In contrast, social norms of masculine behavior among young meat-eating men may act as barriers, particularly in official or business settings or when dining with meat-eating male friends [40*].

Food festivals or gastronomic events emerge as specific types of food environmental structures where consumers may be willing to try APF [20,* 45*]. Visiting food events or festivals may satisfy consumer needs of being adventurous, their curiosity or sensation seeking, which, in turn, are related to stronger intentions to try, greater attractiveness, and willingness to try APF [57, 58]. While restaurants and food festivals/gastronomic events may be the locations where consumers first *try* an APF, the adoption of regular intake of the APF could depend on other structures, such as supermarkets and grocery stores—places where consumers typically buy their food—and the associated barriers and facilitators within these structures [33*].

The findings highlight that certain barriers and facilitators may be relevant in specific types of built food environments where consumers try new foods but do not apply in environments where daily food shopping takes place. For instance, the lack of visible insects and the use of ambiguous names of insect-based APF in restaurants facilitated consumers’ choices, likely by reducing anxiety and increasing the likelihood of trying this type of APF in restaurants [41*]. In contrast, the perceived attractiveness of insect-based APF was low regardless of insect visibility and package labeling of food sold in retail stores.

Our review also provides evidence for differences in facilitators of consumers’ choices to consume plant-based APF and insect-based APF. These differences are particularly evident when considering restaurants as built food environments. Social norms related to masculinity act as barriers to young men visiting vegetarian restaurants or buying and eating plant-based APF in restaurants or at business lunches [40,* 44*]. Previous systematic reviews focusing on individual determinants of insect-based APF have indicated that young men and high sensation seekers are more likely to try eating insects [57, 59]. Therefore, it is possible that social norms regarding masculinity could actually facilitate the consumption of insect-based APF in restaurants among young men.

The results provide no insights into the underlying mechanisms that explain the associations between barriers/facilitators and consumers’ APF choices in specific environmental structures. Unfortunately, such mechanisms were rarely investigated in the original studies. Thus, any discussion of the underlying processes remains hypothetical. It may be assumed that the potential mechanisms may include consumers’ motivations, emotions, and beliefs related to the APF; social and cultural norms about eating novel food; and habits related to buying and preparing any type of food. These mechanisms may be significant (or may be less relevant), depending on the type of physical environmental setting where the choice of APF occurs. For example, the effects of social norms may be stronger in a setting where consuming food is a social activity (e.g., in restaurants) than in a setting where an APF is purchased via a website of an online vendor. Consumers’ curiosity and sensation seeking may be significant drivers for trying new APF in novel environments such as food festivals. In contrast, curiosity may be less important when a consumer’s behavior is aimed at restocking breakfast supplies in a local grocery store. When shopping “on autopilot” (e.g., during routine weekend shopping at a local supermarket), automatic, nonreflective responses to cues may be the key mechanisms determining consumers’ choices. In this context, cues such as the retailer’s strategy of placing a product in a specific section may be among the strongest drivers of consumers’ choices. Future research should explore the common and specific mechanisms determining consumers’ choices across the structures of the built environment.

This study has potential implications for promoting APF choices. Our review lists characteristics of the built food environment to be considered in interventions targeting the initiation and adoption of APF intake. APF promotion strategies may be adjusted to specific types of barriers/facilitators operating in specific structures of the built environment. Furthermore, the results referring to the availability of APF may suggest changes in strategic considerations at the retailer level (for example, a shift from an emphasis on sales via e-commerce to supermarket sales). The results may also inform public health policies and address the physical food environment, helping to prioritize specific changes in the respective setting (e.g., positioning APF in meat and produce sections in supermarkets).

Our study inevitably has certain limitations related to the number, quality, and heterogeneity of the studies included. First, the number of studies we identified was limited, and replications across contexts (e.g., in different countries) are missing. Furthermore, most of the empirical evidence is based on correlation studies; therefore, causal conclusions cannot be drawn. The included research used a broad range of indicators of consumer choices, ranging from intention (to buy or to try/eat) to actual intake. Intention may have a limited effect on the adoption of a new consumer behavior and its maintenance over time [60] because intention is only moderately associated with respective food intake [61]. The quality of 22.3% of the included studies was moderate or low, which limits the reliability of the conclusions. To address publication bias, we conducted additional searches for grey literature (published in outlets other than impacted journals) via searches of the Google Scholar, CORDIS, and ORE databases. Approaches that allow us to quantify the heterogeneity of the results could not be used, as the data were analyzed using a narrative synthesis instead of a meta-analysis.

Future original studies may also need to control for potential confounders (e.g., economic factors such as disposable income in families and food policies operating in the respective countries) and address some methodological shortcomings. For example, specific barriers and facilitators were usually studied in one setting and one location only; hence, the generalizability of the results to other settings (e.g., other supermarket chains) and different locations (e.g., urban versus rural areas) is limited. The characteristics of consumers, such as lower income, may constitute a universal barrier to APF choices [62]. Higher prices of many APF (compared to animal-based protein products) may affect APF affordability among people with lower incomes, regardless of the setting where the purchase takes place. The original research was heterogeneous in terms of the socioeconomic status of the participants, and the effect of socioeconomic variables was usually not considered. Future research should account for the confounding role of economic factors.

Furthermore, the applied methods of the systematic review also have limitations. The use of narrative synthesis and to the inability to conduct a meta-analysis hindered the evaluation of the actual significance and strength of the relations between the characteristics operating in the respective types of built food environments and the indicators of consumers’ choices. Due to very limited empirical evidence for APF other than plant-based and insect-based APF, the proposed extension of the typology for the built environment does not provide insights into the characteristics of the food environment that may promote or hinder mainstreaming krill-based, fungus-based, and bacteria-based alternative proteins or proteins from other sources.

## Conclusions

This review provides novel insights into the barriers and facilitators operating in different types of built food environments that may affect the uptake of novel foods developed with alternative proteins. Barriers and facilitators may refer to the physical characteristics of the structure, food presentation practices, organizational strategies or policies operating in the setting, the actions and beliefs of retailers or consumers while selling or purchasing the APF in the setting, etc. Our results indicate that perceived and actual availability are common facilitators operating across the various types of built environments. The results also indicated several determinants associated with consumers' choices in specific types of built food environments: the ways food is presented in produce sections (supermarkets), consumers’ green and specialty shopping routines (groceries), a mismatch between retailers’ actions of making APF available in one type of environment (e-commerce), and consumers’ preferences for other types of APF environments (supermarkets, groceries). We also indicate that one type of barrier/facilitator operating within a specific type of built food environment may have different associations with consumers’ choices depending on the type of APF (e.g., social norms regarding masculinity as a barrier for plant-based, but not insect-based, APF in restaurants).

### Supplementary Information

Additional file 1: Description of data: The full list of keywords applied in the review; details of data analysis and synthesis; definitions, theoretical background, and categories applied during the coding procedures; description of the included studies; populations’ characteristics in the included studies; Supplementary Table 1 - descriptive information about the original research included into the review

Additional file 2: Description of data: Details of the quality and risk of bias assessment of the included in the systematic review studies

## Data Availability

All data analyzed during this study are either secondary (retrieved from original studies included in the review) or included in this published article (and its supplementary information files).

## Abbreviation

APF: Alternative protein foods
