# Supplementary material for: Characteristics of built food environments associated with alternative protein food choices: a systematic review
Source: Int J Behav Nutr Phys Act. 2024 May 16;21:58. doi: 10.1186/s12966-024-01606-6 (PMC11100026; doi:10.1186/s12966-024-01606-6)
Supplement: Supplementary file 1 — Additional file 1: Description of data: The full list of keywords applied in the review; details of data analysis and synthesis; definitions, theoretical background, and categories applied during the coding procedures; description of the included studies; populations’ characteristics in the included studies; Supplementary Table 1 - descriptive information about the original research included into the review [file 12966_2024_1606_MOESM1_ESM.docx]

**Characteristics of built food environment associated with alternative protein food choices:**

**A systematic review**

**Additional file 1:**

1. The full list of keywords applied in the review
2. Data analysis and synthesis
3. Definitions, theoretical background, and categories applied during the coding procedures
4. Description of the included studies
5. Population characteristics in the included studies

Supplementary Table 1 Descriptive Information about the Original Research Included into the Reviews

1. **The full list of keywords applied in the review**

The search strategy adopted the following groups of keywords:

1. *Alternative protein food:* "cultured meat*" OR "in vitro meat*" OR "synthetic meat*" OR "seaweed*" OR "alga*" OR "insect*" OR "lupin* protein*" OR "dry pea* protein*" OR "chickpea* protein*" OR "cow pea* protein*" OR "pigeon pea* protein*" OR “lentil* protein*” OR “pulse* protein*” OR “legume* protein*” OR “bean* protein*” OR "meat alternative*" OR "meat substitute*" OR "plant-based meat*" OR "meat analogue*" OR “plant-based protein*” OR "rapeseed kernel protein*" OR "mealworm protein*" OR "krill protein*" OR "microbial protein*" OR "cultivated mushroom protein*" OR "fermented fungal protein*" OR "pea protein*" OR "meat analogue*" OR "hybrid meat*" OR "non-meat protein source" OR "novel plant-based alternative*" OR "plant-based protein*" OR "substitute meat protein*" OR "insect-based food*" OR "insect-based protein*" OR "larvae protein*" OR "bacterial protein*" OR "lab grown meat*" OR "cultured meat-based protein*" OR "cultivated meat*" OR "fusarium venenatum" OR "quorn" OR "texture vegetable protein" OR "novel food" OR "innovative food*" OR "alternative type* of food*" OR "alternative protein*" OR "sustainable protein*" OR "soy protein*" OR "mycoprotein*" OR "whey protein*";
2. *Physical built environment:* "home" OR "shop*" OR "retail*" OR "cater*" OR "restaurant*" OR "supermarket" OR "hotel*" OR "farmer market*" OR "grocer*" OR "vendor" OR "kiosk" OR "food environment" OR "school" OR "public institution*" OR "food vend*" OR "built environment" OR "physical environment" OR "food procurement" OR "accommodation" OR "neighborhood*" OR "neighbourhood*" OR "local communit*" OR "urban" OR "local sale" OR "food outlet*" OR "food store*" OR "workplace" OR "rural" OR "suburban" OR "transport" OR "geograph*" OR "architectur*" OR "menu design" OR "canteen" OR "in-store design" OR "point of sale" OR "fast-food store*" OR "fast-casual" OR "local market" OR "buildings" OR “market”
3. *Consumer or behavior-related* : "intake" OR "food" OR "consume*" OR "eat" OR "sale" OR "purchase" OR "buy" OR "sell"*"
4. Data analysis and synthesis

We employed narrative synthesis methods, drawing from the Economic and Social Resesarch Council guidance on narrative synthesis [1,2]. First, a narrative synthesis uses a theoretical model providing the underpinnings for the analyzed patterns of associations [1,2]. In this review, the built environment typology proposed by Downs et al., [3] served as the framework for synthesizing evidence related to associations between built environment characteristics and consumer choices indicators. Second, the preliminary synthesis should be provided, including an initial description of the results of included studies (e.g., their textual description, forming data into a common rubric characterizing the studies, tabulation) [1,2]. Specifically, we coded the studies included along the four categories (type of food product, type of environment, characteristics of the type of environment, and consumer choice indicator) and provided an initial description of the results in the form of table and textual synthesis. The third step accounts for exploring the relationships in the data by examining emerging patterns that allow to identify patterns of associations and provide explanations of differences in the direction of associations. This may be achieved by analyses of emerging cluster groups, conceptual mapping, context description, and frequency distributions [1,2]. In this review, we grouped the studies along the types of the built environment proposed by Downs et al.’s [3] typology. Next, within each built environment type, we clustered the findings referring to plant-based vs. insect-based vs. other types of alternative protein sources. Context factors or specific characteristics of the environment (e.g., ways of exposing food, actual availability at the setting) were used to further examine the patterns of associations. Fourth, the narrative synthesis should account for an assessment of the robustness of the obtained results, for example, using the quality assessment tools that address the respective risk of bias (see ESRC guidance; [1,2]. This review addressed the heterogeneity of studies in reference to the quality of included papers.

1. Definitions, theoretical background, and categories applied during the coding procedures

The t*ypes of alternative protein products* were coded into the following categories, based on protein sources [4]: (1) food developed with land or sea plant-based protein, including food developed with microalgae-based proteins; (2) food including or made of insect-based protein (any type of insects used in the production of food). Within plant-based alternative proteins, we distinguish a specific sub-category of food developed with a combination of plant-based proteins and meat. These products were categorized as plant-meat hybrid foods, including food developed by combining meat products (pork, beef, poultry) with plant protein sources, with a proportion of 50-50% or 25-75% of respective types of sources; (3) food including or made of various types of alternative proteins, including plant-based, insect-based and other types of alternative protein sources, such as krill, bacteria, or fungi.

The *types of structures of* *built food environment* were coded using Downs et al. [3] typology, distinguishing between informal market food environments (mobile vendors, wet markets, kiosks, street vendors) and formal market food environments (supermarkets, hypermarkets, mobile vendors, online vendors, other food retailers/groceries, institutions/public procurement, restaurants, farmer markets).

Where available, we coded the *specific characteristics of the type of food environment*, referring to: the ways the food was exposed in the setting, including, e.g., in the menu, on shelves, side to side versus separately from meat-based protein products; the methods used by the retailers/restaurateurs to promote food from alternative protein sources in a specific food environment; and the social food environment, such as presence of other/specific people in the environment.

The *consumer choice indicators* included three types of variables commonly used in research on behavior determinants, such as the theory of planned behavior [5] or social cognitive theory [6], namely (1) attitudes towards/perceptions of the physical and social environment or the food product itself (i.e., its attractiveness, approval, acceptance, appropriateness), (2) intentions to act, and (3) the actual behavior performance. The indicators of relevant attitudes or perceptions included: acceptability of foods, perceived availability of foods, consumers’ approval or liking of food, and preference for the point of sale (or the type of environment where the food is sold). According to behavior change theories [5,6], attitudes, beliefs, and perceptions may refer to the consumers themselves (e.g., perceived capabilities, skills, or emotions). These types of perceptions, not referring to the physical environment directly, were not considered an indicator of consumers’ choices but rather an individual characteristic of a consumer that determines other consumer choice indicators and, therefore, excluded. The separation of perceptions of/beliefs about the environment from beliefs/perceptions of oneself is used in theoretical approaches focusing on environmental versus individual determinants of other human behaviors (c.f., the model of four domains of active living [7]). The intentions to act included intention to eat (e.g., see behavior change models such as TPB [5]), intention/willingness to pay, and intention/willingness to buy (e.g., [8]). The actual behaviors included: the actual consumption of the food in the study location, the actual purchase by a consumer, sales of a product in the study location/food environment type, and visiting the location selling the alternative protein food products.

1. Description of the included studies

Across the original studies, the following APFs were addressed:

1. *k* = 17 studies discussed the plant-based alternative protein products [9–24]; note: from Vandenbroele et al. [24] only study 1 & 2; see also: Supplementary Table 1);
2. *k* = 15 studies addressed insect-based alternative protein products [19,25–36]; note: from Baker et al. [26] study 1, 2 & 3; from Motoki et al. [19] study 1;
3. *k* = 1 accounted for hybrid meat products (i.e., meat and plant-based meat replacement combined into one product) [16];
4. *k* = 4 analyzed foods from either plant-based proteins or insect-based sources [19,37,38]; from Motoki et al. [19] study 2 & 3;
5. *k* = 3 focused on a broader category of novel food, including either plant-based or insect-based APF, or APF from various sources [37–39].

Regarding the subtypes of environmental structures, none addressed informal market food environment types (wet markets, street vendors, kiosks, mobile vendors), whereas, *k* = 31 (all studies) addressed formal market food environment. In particular:

1. *k* = 6 addressed supermarkets [12,16,17,20,33,36];
2. *k* = 10 addressed other food retailer structures (e.g., groceries) [9,14,23,24,26,28,30,35]; note: from Baker et al. [26] only study 1 & 3; from Vandenbroele et al. [24] only study 1 & 2;
3. *k* = 2 addressed farmer markets [35,39];
4. *k* = 17 addressed restaurants [10,18–23,25–27,29,31,37,38]; note: from Baker et al. [26] only study 2 & 3; from Motoki et al. [19] study 1, 2 & 3;
5. *k* = 2 addressed institutions and public procurement (schools) [11,32];
6. *k* = 4 addressed online vendors [30,34–36];
7. *k* = 1 addressed vending machines [15];
8. *k =* 4 addressed food festivals [19,21]; from Motoki et al. [19] study 1, 2 & 3;
9. No study addressing mobile vendors and hypermarkets was found.

The consumer choices indicators included:

1. Consumers' attitudes and beliefs about, e.g., healthiness and sustainability (*k* = 2 studies; [23,31]);
2. Consumers’ trust and confidence (*k =* 1 study; [36]);
3. Easiness to find APF (*k* = 1 study; [17]);
4. Self-reported indication for the preferred location where the product should be available (*k* = 10 studies; [15,19,21,27,30,35,37,38]; note: from Motoki et al. [19] study 1, 2 & 3);
5. Satisfaction with plant-based proteins (*k =* 1 study; [9]);
6. Consumers approval of a specific cuisine (*k* = 1 study; [22]);
7. Acceptance of APF (*k =* 1 study; [18]);
8. Preferred location for the consumption (*k =* 1 study; [20]);
9. The likelihood to visit a location where APF is sold, (*k =* 1 study; [10]);
10. The intention to (re) visit restaurants (*k =* 1 study; [25]);
11. Consumers’ intention to buy (*k* = 5 studies; (26,28,35); note: from Baker et al. [26] study 1, 2 & 3);
12. Consumers’ intention to pay (*k =* 2 studies; [20,28]);
13. Consumers’ intention to eat (*k* = 6 studies; [13,19,32,33]); note: from Motoki et al. [19] study 1, 2 & 3);
14. Actual sales of products (*k* = 4 studies; [24,34,39]); note: from Vandenbroele et al. [24] only study 1 & 2);
15. Self-reported purchase by the consumers (*k* = 4 studies; [14,20,21,23]).

Studies focusing on actual availability of APF products in the specific structures of built food environment, e.g., schools, were also included (k = 3 studies; [11,12,16]). Although these studies did not address consumer choice indicators (such as e.g., intention to buy or an actual purchase), they provided data regarding a type of built environment and barriers or facilitators operating in this structure.

1. Population characteristics in the included studies

Overall *k* = 31 (86.11%) of the studies included consumer samples from the general population ([9,12,13,15–27,29–31,33–39]; note: from Motoki et al. [19] study 1, 2 & 3; from Vandenbroele et al. [24] only study 1 & 2; from Baker et al. [26] study 1, 2 & 3). Only *k* = 5 enrolled specific populations, such as students, homemakers (women), men only, etc. [10,11,14,28,32]. Original studies were conducted in 22 different countries. Most frequently, the studies were conducted in the United States of America (*n* = 9, 25%), UK (*n* = 4, 11.1%), Belgium (*n=*3, 8.3%), Canada (*n* = 3, 8.3%), Italy (*n =* 3, 8.3%), Germany (*n* =3, 8.3%), Japan (*n*=3, 8.3%). Two studies (5.5%) were conducted in each Australia, France, Netherlands, Spain, and Denmark. Other studies (*n =* 14, 38.8%) were conducted in China, Ireland, Switzerland, Brazil, Portugal, South Korea, Austria, Finland, Norway, and Sweden.

Supplementary Table 1 Descriptive information about the original research included into the reviews

| **First author, year** | **Build Food Environment Structure** | **Population** | **Country** | **Study design & quality index (JBI)** | **Consumer’s choice indicator in studies assessing consumer choices/ Indicators of actual availability in studies addressing the actual presence of APF in the environment (no choice indicators measured in consumers)** | ***Type of alternative proteins*** |
| --- | --- | --- | --- | --- | --- | --- |
|  |  | ***N*; gender; age** |  |  |  | **Associations between the types of environmental structures and APF choices by consumers** |
|  | **Wet markets** | |  |  |  |  |
| No studies identified |  | | | | | |
|  | **Street vendors** | |  |  |  |  |
| No studies identified |  | | | | | |
|  | **Kiosks** | |  |  |  |  |
| No studies identified |  | | | | | |
|  | **Mobile vendors** | |  |  |  |  |
| No studies identified |  | | | | | |
|  | **Supermarkets** | |  |  |  |  |
|  |  | |  |  |  | ***Plant-based alternative proteins*** |
| **Brooker et al. (2022)** [12] |  | Not provided | Australia | Quantitative (Analysis of data from FoodTrack database);  JBI = low | Actual availability in the subtype of the environment | The total number of alternative protein products in supermarkets between 2014 and 2021: 130% increase overall. Subcategories: plant-based meats (150% increase) and legume products (129% increase) increased, but the number of tofu products decreased over time. Across alternative protein products, 58% were available in 1 year only. On average, alternative protein products were available for 2.2 years; 62% of products collected in 2021 had not been collected before (Australia) |
| **Grasso & Jaworska (2020)** [16] |  | Not provided | UK | Qualitative (language analysis)  JBI = high | Actual availability in the subtype of the environment | In total, 38 hybrid (meat combined with plants) products were launched in UK supermarkets in 2016–20, and 12 of these products were available in 2020. The most popular hybrid meat products launched and sold in supermarkets were sausages, with 20 products launched, followed by meatballs (7 products launched), and burgers (6 products launched) |
| **Gravely & Fraser (2018)** [17] |  | *N* = 24 participants | Canada | Qualitative (interviews)  JBI = low | Easiness to find APF  Actual availability in the subtype of the environment | Shelf space in meters: supermarkets allocate a significantly higher amount of shelf space for animal-based protein (*M* = 131.9 m) compared to plant-based protein (*M* = 30.8 m)  Significantly more sales per meter shelf space within supermarkets for animal-based protein products (*M* = 0.71) than plant-based protein products (*M* = 0.36)  A significantly higher proportion of animal-based protein products had sale and/or descriptive signage displayed at their point-of-purchase display (*M* = 32%) compared to plant-based protein products (*M* = 2%)  Participants agreed that the meat, seafood, and dairy sections were the most “prominent” in-store, meaning they occupy the most space and are equipped with significant promotional signage advertising sales and/ or the quality of products. For the plant-based protein, consumers noted how many of the products were “hidden” around the store.  The most common area for plant-based protein was in the grocery aisles of supermarkets (34%), although this area was disproportionately represented by packaged grains, legumes, nuts, and seeds. Plant-based meat substitutes were commonly clustered in the produce (i.e., fruits and vegetables) section of the store (44%), and plant-based dairy products were most commonly found in the dairy section of the store (41%). Aside from the produce section, the organics section was another location where plant-based proteins were clustered, although this was most important for the high-end conventional retail stores that had an expansive natural values section containing a high number of organic and premium products.  Only about a third of the plant-based meat substitutes were placed on the same shelving unit as other meat products.  The organization of plant-based proteins on the shopping floor was related to participant perceptions about the ease of locating these products. Participants rated it easier on a Likert scale to find animal-based protein (*M* = 4.67, *SD* = 0.65) than plant-based protein (*M* = 3.64, *SD* = 1.14, *p* < .01). The most common factor that participants communicated in determining the ease of finding a product was “consistency” in product location. Consistency relates to whether a product can be located between stores in the same section on the shopping floor.  Several consumers indicated that plant-based sources of protein, especially plant-based meat analogues, were often not in the sections that they expected them to be and had difficulty finding these products in new shopping environments.  By this, retailers want to “keep ahead” of consumers in anticipation of a rising demand for plant-based protein, but are simultaneously highly attentive to the engagement level of consumers.  Supermarkets want to appear progressive by catering to the diverse tastes of their customers, but they are only willing to take a certain degree of risk in “pushing” new products onto consumers (slim profit margin, low shelf life) when innovating new plant-based meat substitute products, there’s a “prove it to me first” mentality on the part of the supermarket. This finding underscores supermarkets, as relatively risk-averse entities that require sufficient demand signals before they are willing to incorporate more plant-based protein products onto their shelves.  Sensitivity towards product organization: Keeping plant-based meat substitutes away from meat to satisfy the perceived concerns of vegetarians and vegans.  Reserving prime retail space for “core” items like meat and dairy (including alternative proteins there would reduce retail incomes). Organizing plant-based protein in the natural values section to profit from price premiums. |
| **Ortega et al. (2022)** [20] |  | *N* = 2015 participants; 51% women; *M*_age_ = 35 years old | China | Quantitative (online survey)  JBI = low | Preferred location for the consumption  Intention to pay | Plant-based protein: rather than at home, consumers preferred to eat APF away from home locations (e.g., restaurants, bars) or in supermarkets.  Consumers were willing to pay more for APF, whereas those who usually shopped in cheaper domestic discount supermarkets indicated they would pay less for APF, which may be explained by the perceived higher quality of the products available in international chains. |
|  |  |  |  |  |  | ***Insect-based alternative proteins*** |
| **Menozzi et al. (2017)** [33] |  | *N* = 109 participants; 61.9% women; *M*_age_ = 23.6 years old | Italy | Quantitative (survey, semi-structured questionnaire, tasting session, content analysis, TPB questionnaire)  JBI = low | Intention to eat | Perceived lack of availability in the supermarkets was a barrier, reducing the intention to eat a product containing insect flour. |
| **Reverberi (2021)** [36] |  | Not provided | Not provided | Qualitative (interviews)  JBI = high | Trust and confidence | Packaged processed insect (PPI)- based products. The interviewed companies confirmed that insect-based products perform better through physical retailers than e-commerce. Between 2015 and 2018, start-ups focused on e-commerce. PPIs still need to win consumer trust. The best approach is an exposure on the shelves of supermarkets or corner shops. However, traditional food retailers expect products to sell quickly, which might be challenging. But, obtaining shelf exposure will improve consumers’ emotional confidence in PPIs. Consumers tend to trust a supermarket chain more than an online shop. Until PPIs are widely distributed in normal retail points of purchase, consumers will not fully embrace them as credible and safe. |
|  | **Hypermarkets** | |  |  |  |  |
| No studies identified |  | | | | | |
|  | **Other retailers (groceries)** | |  |  |  |  |
|  |  |  |  |  |  | ***Plant-based alternative proteins*** |
| **Drake & Gerard (2003)** [14] |  | Participants recruited from university campus: *n* = 388; 45% women; Participants recruited in a grocery store: *n* = 159; 77% women  Age range between 18 and 45 years old | USA (Mississippi) | Quantitative (experiment)  JBI = low | Self-reported purchases by the consumers | Higher intake among students who purchase food in groceries compared to those who buy their food on university campuses |
| **Weinrich & Elshiewy (2023)** [23] |  | *N* = 938 participants; 51% women; Age range between 16 and >56 years old | Germany, France, Netherlands | Quantitative (survey)  JBI = low | Consumers’ attitudes and beliefs (e.g., about healthiness, sustainability, etc.);  Self-reported purchase by the consumers | Perceiving microalgae-based food as healthy, sustainable, and nutritious was unrelated to habits of shopping in specialty food stores among consumers from France, Germany, and the Netherlands (men and women subsamples ). |
| **Vandenbroele et al. (2021)**  **(2 studies)** [24] |  | Study 1: *N* <100,000; Gender not provided  Study 2: *N* = 231 participants;  Study 1: Gender not provided  Study 2: *n* = 111 males | Belgium | Quantitative (field experiment & experiment 2x2 mixed models design)  JBI = low | Actual sales of products | Presentation of plant-based alternative foods in large groceries  In the month preceding the intervention, which was used as a pre-intervention period, the meat product was offered in the butchery, and the meat substitute was available on a separate, vegetarian shelf in the vegetables and fruits department. This pre-intervention period reflects the default choice architecture. Then, during the one-month intervention period, the meat substitute remained on the vegetarian shelf but also appeared in the butchery, pairwise with the meat product and in proximity to other sandwich offerings containing meat. No intervention took place in the eight control stores; the meat and meat substitute options were offered separately. The control stores were all branches of the same retail chain, with similar layouts and assortments. The control stores' data helped rule out environmental influences (e.g., promotions of products in the same category). Meat substitute sales were higher in the experimental store during the intervention period.  The sandwich department in the hypothetical retail store, established in a university lab, consisted of two refrigerators, placed back-to-back; only one was immediately visible to the participants as they entered the lab. In each condition, an equal number of products appeared in both fridges. All meat substitutes were presented next to their meat-based alternative, such that each fridge contained two pairs. In the control condition, the meat and meat substitutes were in different fridges.  Four products were placed in the immediately visible fridge to create a high product visibility condition. The four remaining products, situated in the fridge that was not immediately visible, represented the low visibility condition. Accordingly, visibility was not a between-subjects factor because all participants were exposed to eight products, of which four were high in visibility and four others were low in visibility.  Main effect of visibility: Higher visibility led to more purchases of meat substitutes. The main effect of the pairwise presentation was also significant, with more meat substitutes sold in such a presentation mode |
| **Aaslyng & Højer (2021)** [9] |  | *N* = 395 participants; 78% women;  Most participants were between 18 and 29 years old | Denmark | Quantitative (online survey)  JBI = low | Satisfaction with plant-based proteins | Perceived availability of plant-based alternatives in groceries where food is usually purchased was related to satisfaction with plant-based proteins. |
| **Clark & Bogdan (2019)** [13] |  | *N* = 410 participants; 55.1% women;  Most participants were between 55 and 75 years old | Canada | Quantitative (online survey)  JBI = low | Intention to eat  Intention to buy | When asked about the key barriers to trying new plant-based alternative proteins, 25% of respondents indicated “not available where I buy food.” Also, those who intended to buy such food claimed that the availability was low. While 42.7% of respondents likely to purchase new protein alternatives identified the lack of availability as a factor in their decision-making, this was statistically higher when compared to respondents who were undecided (19.5%) or unlikely to buy them (10.1%). |
|  |  |  |  |  |  | ***Insect-based alternative proteins*** |
| **Baker et al. (2016)**  **(3 studies)** [26] |  | Study 1: *N* = 221 participants  Study 2: *N* = 200 participants  Study 3: *N* = 201 participants  Study 1: 43% women  Study 2: 38% women  Study 3: 47.8% women  *M*_age_ = 35 years old | USA | Quantitative (experiment)  JBI = low | Intention to buy | No major differences in intention to purchase, expected liking/ attractiveness: Products in a grocery with visible insects on the package vs. pictures of insect-based powder + a name in Latin. |
| **Collins et al. (2019)** [28] |  | *N* = 1020;  65% women in online survey;  Age range between 12 and 90 years old | UK | Quantitative (survey)  JBI = low | Intention to buy;  Intention to pay | Regular green shoppers were more likely to choose food with insects invisible. High frequency of green shopping was a predictor of willingness to pay for insect-based foods |
| **Herbert & Beacom (2021)** [30] |  | *N* = 105 survey respondents;  Age range between 18 and 62 years old | Ireland | Mixed methods (focus groups, survey)  JBI = low | Self-reported indication for the preferred location where the product should be available | 53% (the largest group) indicated that insect-based products should be sold in convenience stores/petrol stations. |
| **Porretta et al. (2019)** [35] |  | *N* = 106 participants; *n* = 71 women; | USA and Canada | Quantitative (experiment)  JBI = high | Self-reported indication for the preferred location where the product should be available;  Intention to buy | People > 50 years old were more likely to buy insect-based food if it was available in many kinds of food stores/retail (relevant for men only) |
|  | **Farmer’s markets** | |  |  |  |  |
|  |  |  |  |  |  | ***Insect-based alternative protein food*** |
| **Porretta et al. (2019)** [35] |  | *N* = 106 participants; *n* = 71 women | USA and Canada | Quantitative (experiment)  JBI = high | Self-reported indication for the preferred location where the product should be available;  Intention to buy | People > 50 years old were more likely to buy insect-based food if it was available from local producers in small farmer’s markets. |
|  |  |  |  |  |  | ***Various novel alternative protein food, including insect-based and plant-based alternative proteins, or alternative proteins from any other sources (e.g., fungi, bacteria)*** |
| **Aerni et al. (2011)** [39] |  | *N* = 3275 participants | Switzerland (Zurich and Lausanne) | Quantitative (experiment)  JBI = low | Actual sales of products | Genetically modified novel food based on corn: Lower sales at a local food market/farmers market in the center of the city than at the railway stations [in Zurich and Lausanne, Switzerland]. |
|  | **Restaurants** | |  |  |  |  |
|  |  |  |  |  |  | ***Plant-based alternative protein food*** |
| **Bogueva et al. (2022)** [10] |  | *N* = 36 participants; males only  (Generation Z and Millenials) | Australia | Qualitative (semi-structured in-depth interviews)  JBI = low | Likelihood of visiting a location where APF is sold | Young daily meat-eating men: Vegan burgers are seen as a new trend for which people are prepared to stay in a queue to have that novel experience (social pressure – presenting oneself as being a part of this new experience) “happy to line up with other men.” “restaurants serving plant-based alternatives are becoming an arena of social change and an important turning point in the transition toward making more ethical and sustainable food choices while connecting with the people you love.” However, “Sharing food selfies on social media from the vegan restaurant is seen as a formula for trouble and irreversible image destruction.” (it’s all about masculinity) |
| **Michel (2020)** [18] |  | *N* = 1039 participants; 51% women; Age range between 20 and 69 years old | Germany | Quantitative (survey)  JBI = low | Acceptability | For omnivores and flexitarians, the acceptance ratings for eating plant-based meat alternatives during business lunches at restaurants were low. |
| **Ortega et al. (2022)** [20] |  | *N* = 2015 participants; 51% women; *M*_age_ = 35 years old | China | Quantitative (online survey)  JBI = low | Self-reported purchase by the consumers | This study found a similar intake of alternative protein-based food in establishments for food away from home consumption (e.g., restaurants) vs. at home vs. at supermarkets.  Similar purchase and intake of alternative protein-based food in establishments for food away from home consumption (e.g., restaurants) vs. at home. Consumption that takes place in away-from-home food establishments (in China), which are often seen as an extension of at-home cuisine; and it's further fueled by the recent increase in the availability of alternative meat products in restaurant settings. |
| **Palmieri & Forleo (2021)** [21] |  | *N* = 257 participants; *n*= 148 women; *M*_age_ = 36 years old | Italy | Quantitative (web-based survey)  JBI = low | Self-reported purchase by the consumers | Those who have the opportunity to find it on a menu are more likely to purchase and consume algae-based food |
| **Perez-Llorens (2020)** [22] |  | Not provided | Not provided | Qualitative (overview)  JBI = moderate | Consumers approval of a specific cuisine | The world’s most-celebrated chefs have ‘discovered’ or ‘rediscovered’ microalgae and their potential to be included as ingredients in their cuisine. Microalgae have been incorporated in their menus for a growing audience of consumers voracious for novelty and who identify with the chef’s discourses and philosophy about sustainability, ethnicity, authenticity, or exotic nature. As has already happened with seaweeds, avant-garde restaurants can popularize the use of microalgae as an additional ingredient in casual or mid-range restaurants as well as home cuisine |
| **Weinrich & Elshiewy (2023)** [23] |  | *N* = 938 participants; 51% women; Age range between 16 and >56 years old | Germany, France, Netherlands | Quantitative (survey)  JBI = low | Consumers attitudes and beliefs (e.g., about healthiness, sustainability) | Perceiving microalgae-based food as healthy, sustainable, and nutritious was related to more frequent dining out/going to restaurants with friends and family among Dutch women, but it was related to less frequent dining out with family and friends among Dutch men and German men. No association were observed for French consumers, both men and women, nor for German women |
|  |  |  |  |  |  | ***Insect-based alternative protein food*** |
| **Ali & Ali (2022)** [25] |  | *N* = 702 participants; 37.7% women; Age range between 26 and 35 years old | USA | Quantitative (online survey)  JBI = low | Intention to (re) visit restaurants | Low risk perception (e.g., getting ill) and low tension/anxiety while eating respective types of foods related to higher intention to visit restaurants |
| **Baker et al. (2016)** [26] |  | Study 1: *N* = 221 participants  Study 2: *N* = 200 participants  Study 3: *N* = 201 participants  Study 1: 43% women  Study 2: 38% women  Study 3: 47.8% women  *M*_age_ = 35 years old | USA | Quantitative (experiment)  JBI = low | Intention to buy | Restaurant customers indicated a preference for insects being invisible: lower intention to purchase, lower expected liking, and lower attractiveness were found for food with visible insects compared to food without visible insects and a vague description of insect-based ingredients.  A lack of visible insects in a restaurant served meal was related to low perceived risks |
| **Bisconsin Junior et al. (2022)** [27] |  | *N* = 780 participants; 52.3% women; Age range between 18 and > 51 | Brazil | Qualitative (interviews)  JBI = low | Self-reported indication for the preferred location where the product should be available | Open-ended questions regarding “how would you eat food made with edible insects?”. The majority states – “with an expert”, followed by: “in a restaurant”, and “with someone who knows how to prepare it” |
| **Florença et al. (2021)** [29] |  | *N* = 213 participants; 79% women; Age range between 18 and > 66 years old | Portugal | Quantitative (online questionnaire)  JBI = low | Actual availability in the subtype of the environment, e.g., restaurants | “Some European gourmet restaurants use edible insects in culinary preparation” – only 31% agree |
| **Hwang et al. (2020)** [31] |  | *N* = 448 participants; 49.8% women; *M*_age_ = 38 years old | South Korea | Quantitative (web-based questionnaire)  JBI = low | Consumers attitudes and beliefs (e.g., about healthiness, sustainability) | Being an environmental advocate (ability to convince others to act for environment conservation) was related to a better image of the insect restaurants. A positive image of edible insect restaurants was related to a higher intention to eat insects |
|  |  |  |  |  |  | ***Various novel alternative protein food, including insect-based and plant-based alternative proteins, or alternative proteins from any other sources (e.g., fungi, bacteria)*** |
| **Cai et al. (2021)** [37] |  | *N* = 20 participants; 20% women | USA | Qualitative (in-depth interviews)  JBI = low | Self-reported indication for the preferred location where the product should be available | - Restaurant image building (destination for sustainable leisure and entertainment, growing media recognition)  - Restaurant promotion (attractiveness of pushing boundaries with unusual ingredients, encouraging customers to try novel food (including various types of APF) in small portions; no charges if the meal is not satisfactory; online demonstrations by chefs; promotional strategies giving customers a greater sense of freedom of choice);  - Name ambiguity of meals with insects (language toning down unusual origin, e.g., insects);  - Deliberate beautification in presentation – garnishing to obscure ingredients and reduce neophobic tendencies |
| **Schwark et al. (2020)** [38] |  | First Round: *N* = 60 participants  Second Round: *N* = 22 participants  First Round: 81.7% males, 10% women;  Second Round: 90.9% males, 9.1% women | Asia, Europe, South America, North America | Quantitative (questionnaire)  JBI = moderate | Self-reported indication for the preferred location where the product should be available | Alternative proteins (insect and plant-based): Haute cuisine restaurant trends predictions by expert panels (selected based on their entry on the official Guide Michelins website).  It is predicted that vegetable and insect-based proteins will be served rather than in vitro/cultivated meat. The experts expect that the trend towards local food will continue and that ingredients from distant regions will not play a more important role |
| **Motoki et al. (2022)**  **(3 studies)** [19] |  | Study 1: *N* = 117 participants;  Study 2: *N* = 108 participants;  Study 3 = 120 participants  Study 1: *n* = 47 women;  Study 2: *n* = 46 women;  Study 3: *n* = 56 women  *M*_age_= 41 years old | Japan | Quantitative (experiment)  JBI = low | Intention to eat | Plant-based and insect-based meat replacements – higher willingness to try at a food festival, followed by a restaurant (lower at home, café, bar, pub) |
|  | **Institutions and public procurement: schools** | | | |  |  |
| **Borkowski et al. (2020)** [11] |  | Not provided | USA (New York) | Quantitative  JBI = low | Actual availability in the subtype of the environment, e.g., schools | Number of times plant-based meat alternatives were provided for lunch at schools (kindergarten to 8^th^ grade): In public schools, they were not offered (hot breakfast or lunches), while meat was available every day. In private schools, they were offered an average of 0.7 days out of 5 days per week (with various types of meat offered on the remaining days) |
|  |  |  |  |  |  | ***Insect-based alternative protein food*** |
| **Jones (2020)** [32] |  | *N* = 187 participants; Age range between 7 and 14 years old | UK | Mixed methods (pre- and post- questionnaire, interview, observation during workshops)  JBI = moderate | Intention to eat | Workshop at schools (45 min) with an entomologist and a chef discussing reasons for insect consumption in the West, pros/cons, and featuring the tasting of two products. Pre-workshop self-reported willingness to choose insects for school lunch stood at 18-35%, while post-workshop, it increased to 45-60% (at least a 20% rise). |
|  | **Mobile vendors** | |  |  |  | **-** |
| No studies identified |  | | | | | |
|  | **Online vendors** | |  |  |  |  |
|  |  |  |  |  |  | ***Insect-based alternative protein food*** |
| **Reverberi (2021)** [36] |  | Not provided | Not provided | Qualitative (interviews)  JBI = high | Trust and confidence | Packaged processed insect (PPI)- based products. The interviewed companies confirmed that insect-based products perform better through physical retailers than e-commerce. Between 2015 and 2018, start-ups focused on e-commerce. PPIs still need to win consumer trust. The best approach is an exposure on the shelves of supermarkets or corner shops. However, traditional food retailers expect products to sell quickly, which might be challenging. But, obtaining shelf exposure will improve consumers’ emotional confidence in PPIs. Consumers tend to trust a supermarket chain more than an online shop. Until PPIs are widely distributed in normal retail points of purchase, consumers will not fully embrace them as credible and safe |
| **Pippinato et al. (2020)** [34] |  | Not provided | Austria, Belgium, Denmark, Finland, France, Germany, Italy, The Netherlands, Norway, Spain, Sweden, UK | Quantitative (survey)  JBI = high | Actual sales of products | Edible insect producers identified in 12 European countries primarily distribute their products through e-commerce (n = 48), with physical sales at farms, catering, and restaurants being less common (n =9). The total number of companies providing physical sales: n =11. |
| **Herbert & Beacom (2021)** [30] |  | *N* = 15 focus groups  *N* = 105 survey respondents  Age range = 18-62 | Ireland | Mixed methods (focus groups, survey)  JBI = low | Self-reported indication for the preferred location where the product should be available | 53 % (the largest group) indicated that insect-based products should be sold in convenience stores/petrol stations |
| **Porretta et al. (2019)** [35] |  | *N* = 106 participants; *n* = 71 women; | USA and Canada | Quantitative (experiment)  JBI = high | Self-reported indication for the preferred location where the product should be available | People > 50 years old were more likely to buy insect-based food if they were available in many kinds of food stores/retail (relevant for men only) |
|  | **Food festivals** | |  |  |  |  |
|  |  |  |  |  |  | ***Various novel alternative protein food including insect-based and plant-based alternative proteins, or alternative proteins from any other sources (e.g., fungi, bacteria)*** |
| **Motoki et al. (2022)** [19] |  | Study 1: *N* = 117 participants,  Study 2: *N* = 108 participants  Study 3: *N* = 120 participants  Study 1: *n* = 47 women  Study 2: *n* = 46 women  Study 3: *n* = 56 women  *M*_age_= 41 years old | Japan | Quantitative (experiment)  JBI = low | Intention to eat  Self-reported indication for the preferred location where the product should be available | Potential consumers asked about their preferred location to try insect-based food or novel food with plant-based alternative proteins reported that they are most willing to try it during a food festival, whereas significantly lower levels were reported for trying at home, restaurant, café, bar, or pub. |
|  |  |  |  |  |  | ***Plant-based alternative protein food*** |
| **Palmieri & Forleo (2021)** [21] |  | *N* = 257; *N* = 148 women; *M*_age_ = 36 years old | Italy | Quantitative (web-based survey)  JBI = low | Self-reported indication for the preferred location where the product should be available  Self-reported purchase by the consumers | Consumers who reported an opportunity to consume seaweed during a gastronomic event or a trip are more likely to buy and consume algae-based foods than those who did not participate in such events |
|  | **Vending machines** | |  |  |  |  |
|  |  |  |  |  |  | ***Plant-based alternative protein food*** |
| **Garcia-Segovia et al. (2020)** [15] |  | *N* = 85; 27.1% women; Age range between 25 and 69 years old | Spain | Quantitative (pre-and post- questionnaire)  JBI = low | Self-reported indication for the preferred location where the product should be available | Algae-based breadstick Participants indicated that it should be eaten as a vegetarian snack, sold from the vending machine (it was not perceived as a meal substitute or to be eaten with specific food only) |

Note: Study design = Type of the study; JBI = Joanna Briggs Institute overall study quality index; Study quality values are reported as three levels of risk of bias: low risk, moderate risk, or high risk; APF = alternative protein food

**References:**

1. Campbell M, Katikireddi SV, Sowden A, Thomson H. Lack of transparency in reporting narrative synthesis of quantitative data: a methodological assessment of systematic reviews. J Clin Epidemiol. 2019;105:1–9.

2. Popay J, Roberts H, Sowden A, Petticrew M, Arai L, Rodgers M, et al. Guidance on the conduct of narrative synthesis in systematic reviews: A product from the ESRC Methods Programme. 2006.

3. Downs SM, Ahmed S, Fanzo J, Herforth A. Food Environment Typology: Advancing an Expanded Definition, Framework, and Methodological Approach for Improved Characterization of Wild, Cultivated, and Built Food Environments toward Sustainable Diets. Foods Basel Switz. 2020;9(4):532.

4. Grossmann L, Weiss J. Alternative Protein Sources as Technofunctional Food Ingredients. Annu Rev Food Sci Technol 2021;12(1):93–117.

5. Ajzen I, Schmidt P. Changing Behavior Using the Theory of Planned Behavior. In: Hamilton K, Cameron LD, Hagger MS, Hankonen N, Lintunen T, editors. The Handbook of Behavior Chang. Cambridge: Cambridge University Press; 2020. p. 17–31.

6. Luszczynska A, Schwarzer R. Changing Behavior Using Social Cognitive Theory. In: Hamilton K, Cameron LD, Hagger MS, Hankonen N, Lintunen T, editors. The Handbook of Behavior Change. Cambridge: Cambridge University Press; 2020. p. 32–45.

7. Sallis JF, Cervero RB, Ascher W, Henderson KA, Kraft MK, Kerr J. An Ecological Approach to Creating Active Living Communities. Annu Rev Public Health. 2006;27(1):297–322.

8. Lu ZY, Hsee CK. Less willing to pay but more willing to buy: How the elicitation method impacts the valuation of a promotion. J Behav Decis Mak. 2019;32(3):334–45.

9. Aaslyng MD, Højer R. Introducing Tempeh as a New Plant-Based Protein Food Item on the Danish Market. Foods. 2021;10(11):2865.

10. Bogueva D, Marinova D, Bryant C. Meat Me Halfway: Sydney Meat-Loving Men’s Restaurant Experience with Alternative Plant-Based Proteins. Sustainability. 2022;14(3):1290.

11. Borkowski S, Rubenstein W, Galvez M, Deierlein AL. Prevalence of meats offered during meals at New York city schools. Health Behav Policy Rev. 2020;7(2):146–53.

12. Brooker PG, Hendrie GA, Anastasiou K, Colgrave ML. The range and nutrient profile of alternative protein products sold in Australian supermarkets between 2014 and 2021. Int J Food Sci Nutr. 2022;73(8):1067–79.

13. Clark LF, Bogdan AM. The Role of Plant-Based Foods in Canadian Diets: A Survey Examining Food Choices, Motivations and Dietary Identity. J Food Prod Mark. 2019;25(4):355–77.

14. Drake M a., Gerard P d. Consumer Attitudes and Acceptability of Soy-fortified Yogurts. J Food Sci. 2003;68(3):1118–22.

15. García-Segovia P, García Alcaraz V, Tárrega A, Martínez-Monzó J. Consumer perception and acceptability of microalgae based breadstick. Food Sci Technol Int Cienc Tecnol Los Aliment Int. 2020;26(6):493–502.

16. Grasso S, Jaworska S. Part Meat and Part Plant: Are Hybrid Meat Products Fad or Future? Foods. 2020;9(12):1888.

17. Gravely E, Fraser E. Transitions on the shopping floor: Investigating the role of Canadian supermarkets in alternative protein consumption. Appetite. 2018;130:146–56.

18. Michel F, Hartmann C, Siegrist M. Consumers’ associations, perceptions and acceptance of meat and plant-based meat alternatives. Food Qual Prefer. 2021;87:104063.

19. Motoki K, Park J, Spence C, Velasco C. Contextual acceptance of novel and unfamiliar foods: Insects, cultured meat, plant-based meat alternatives, and 3D printed foods. Food Qual Prefer. 2022;96:104368.

20. Ortega DL, Sun J, Lin W. Identity labels as an instrument to reduce meat demand and encourage consumption of plant based and cultured meat alternatives in China. Food Policy. 2022;111:102307.

21. Palmieri N, Forleo MB. An Explorative Study of Key Factors Driving Italian Consumers’ Willingness to Eat Edible Seaweed. J Int Food Agribus Mark. 2022;34(4):433–55.

22. Pérez-Lloréns JL. Microalgae: From staple foodstuff to avant-garde cuisine. Int J Gastron Food Sci. 2020;21:100221.

23. Weinrich R, Elshiewy O. A cross-country analysis of how food-related lifestyles impact consumers’ attitudes towards microalgae consumption. Algal Res. 2023;70:102999.

24. Vandenbroele J, Slabbinck H, Van Kerckhove A, Vermeir I. Mock meat in the butchery: Nudging consumers toward meat substitutes. Organ Behav Hum Decis Process. 2021;163:105–16.

25. Ali L, Ali F. Perceived risks related to unconventional restaurants: A perspective from edible insects and live seafood restaurants. Food Control. 2022;131:108471.

26. Baker MA, Shin JT, Kim YW. An Exploration and Investigation of Edible Insect Consumption: The Impacts of Image and Description on Risk Perceptions and Purchase Intent. Psychol Mark. 2016;33(2):94–112.

27. Bisconsin-Júnior A, Rodrigues H, Behrens JH, da Silva MAAP, Mariutti LRB. “Food made with edible insects”: Exploring the social representation of entomophagy where it is unfamiliar. Appetite. 2022;173:106001.

28. Collins CM, Vaskou P, Kountouris Y. Insect Food Products in the Western World: Assessing the Potential of a New ‘Green’ Market. Ann Entomol Soc Am. 2019;112(6):518–28.

29. Florença SG, Correia PMR, Costa CA, Guiné RPF. Edible Insects: Preliminary Study about Perceptions, Attitudes, and Knowledge on a Sample of Portuguese Citizens. Foods. 2021;10(4):709.

30. Herbert M, Beacom E. Exploring Consumer Acceptance of Insect-based Snack Products in Ireland. J Food Prod Mark. 2021;27(6):267–90.

31. Hwang J, Choe JY (Jacey), Kim JJ. Strategy for enhancing the image of edible insect restaurants: Focus on internal environmental locus of control. J Hosp Tour Manag. 2020;45:48–57.

32. Jones V. ‘Just don’t tell them what’s in it’: Ethics, edible insects and sustainable food choice in schools. Br Educ Res J. 2020;46(4):894–908.

33. Menozzi D, Sogari G, Veneziani M, Simoni E, Mora C. Eating novel foods: An application of the Theory of Planned Behaviour to predict the consumption of an insect-based product. Food Qual Prefer. 2017;59:27–34.

34. Pippinato L, Gasco L, Vita GD, Mancuso T. Current scenario in the European edible-insect industry: a preliminary study. J Insects Food Feed. 2020;6(4):371–81.

35. Porretta S, Gere A, Radványi D, Moskowitz H. Mind Genomics (Conjoint Analysis): The new concept research in the analysis of consumer behaviour and choice. Trends Food Sci Technol. 2019;84:29–33.

36. Reverberi M. The new packaged food products containing insects as an ingredient. J Insects Food Feed. 2021;7(5):901–8.

37. Cai CH, Ding A, Legendre TS. Exploring persuasive sales techniques to improve customer acceptance of sustainable but unfamiliar menu in restaurants. Int J Contemp Hosp Manag. 2021;33(10):3093–114.

38. Schwark N, Tiberius V, Fabro M. How Will We Dine? Prospective Shifts in International Haute Cuisine and Innovation beyond Kitchen and Plate. Foods. 2020;9(10):1369.

39. Aerni P, Scholderer J, Ermen D. How would Swiss consumers decide if they had freedom of choice? Evidence from a field study with organic, conventional and GM corn bread. Food Policy. 2011;36(6):830–8.
