# Supplementary material for: Characteristics of built food environments associated with alternative protein food choices: a systematic review
Source: Int J Behav Nutr Phys Act. 2024 May 16;21:58. doi: 10.1186/s12966-024-01606-6 (PMC11100026; doi:10.1186/s12966-024-01606-6)
Supplement: Supplementary file 2 — Additional file 2: Description of data: Details of the quality and risk of bias assessment of the included in the systematic review studies [file 12966_2024_1606_MOESM2_ESM.pdf]

Characteristics of built food environment associated with alternative protein food choices:
A systematic review

Additional file 2:

Supplementary Table 2a The results of quality evaluation and risk of bias assessment performed with JBI tool for quantitative studies

Supplementary Table 2b The results of quality evaluation and risk of bias assessment performed with JBI tool for qualitative studies

Supplementary Table 2a The results of quality evaluation and risk of bias assessment performed with JBI tool for quantitative studies

No.	Publication	1. Were the criteria for inclusion in the sample clearly defined?	2. Were the study participants and the setting described in detail?	3. Was the predictor measured in a valid and reliable way	4. Were objective, standard criteria used for measurement of the condition?	5. Were confounding factors identified?	6. Were strategies to deal with confounding factors stated?	7. Were the outcomes measured in a valid and reliable way?	8. Was appropriate statistical analysis used?	No. of 'YES'	No. of 'YES'*2 + No. of 'UN'*1	Overall quality score	Overall risk of bias
		Final Score	Final Score	Final Score	Final Score	Final Score	Final Score	Final Score	Final Score			% of maximum score (in case of 'NA' the maximum score was adjusted)	LOW ≥ 70%; MODERATE = 50-69%; HIGH ≤ 49%
1	Aaslyng [2021]	Yes	Yes	Yes	Yes	No	No	Yes	Yes	6	12	75	LOW
2	Aerni [2011]	Yes	Yes	Yes	Yes	Yes	Yes	Yes	Yes	8	16	100	LOW
3	Ali [2022]	Yes	Yes	Yes	Yes	No	Yes	Yes	Yes	7	14	88	LOW
4	Baker [2016]	No	Yes	Yes	Yes	Yes	Yes	Yes	Yes	7	14	88	LOW
5	Borkowski [2020]	Yes	Yes	NA	NA	Yes	No	Yes	Yes	5	10	83	LOW
6	Brooker [2022]	Yes	Yes	NA	NA	Yes	Yes	Yes	Yes	6	12	100	LOW
7	Clark [2019]	Yes	Yes	Yes	Yes	Yes	Yes	Yes	Yes	8	16	100	LOW
8	Collins [2019]	Yes	Yes	Yes	Yes	Yes	Yes	Yes	Yes	8	16	100	LOW
9	Drake [2003]	Yes	Yes	Yes	Yes	No	No	Yes	Yes	6	12	75	LOW
10	Florença [2021]	Yes	Yes	Yes	Yes	Yes	Yes	Yes	Yes	8	16	100	LOW
11	García-Segovia [2020]	No	No	Yes	Yes	Yes	Yes	Yes	Yes	6	12	75	LOW
12	Herbert [2021]	No	Yes	Yes	Yes	Yes	No	Yes	Yes	6	12	75	LOW
13	Hwang [2020]	Yes	Yes	Yes	Yes	Yes	Yes	Yes	Yes	8	16	100	LOW
14	Jones [2020]	Yes	Yes	NA	NA	NA	NA	UN	No	2	5	63	MODERATE
15	Menozzi [2017]	Yes	Yes	Yes	Yes	Yes	Yes	Yes	Yes	8	16	100	LOW
16	Michel [2021]	Yes	Yes	Yes	Yes	Yes	Yes	Yes	Yes	8	16	100	LOW
17	Motoki [2022]	Yes	Yes	Yes	Yes	Yes	Yes	Yes	Yes	8	16	100	LOW
18	Ortega [2022]	Yes	Yes	Yes	Yes	Yes	Yes	Yes	Yes	8	16	100	LOW
19	Palmieri [2021]	Yes	Yes	Yes	Yes	Yes	Yes	Yes	Yes	8	16	100	LOW
20	Pippinato [2020]	Yes	No	NA	NA	No	No	Yes	NA	2	4	40	HIGH
21	Porretta [2018]	No	No	Yes	Yes	No	No	Yes	No	3	6	38	HIGH
22	Schwark [2020]	Yes	Yes	Yes	Yes	No	No	Yes	No	5	10	63	MODERATE
23	Vandenbroele [2021]	Yes	Yes	Yes	Yes	Yes	Yes	Yes	Yes	8	16	100	LOW
24	Weinrich [2023]	Yes	Yes	Yes	Yes	Yes	Yes	Yes	Yes	8	16	100	LOW

Note:
UN - unclear
NA - not applicable

Yes - 2 points
Unclear - 1 point
No - 0 points
In case of NA - the maximum score was adjusted respectively
Maximum score (when all criteria were rated 'Yes') = 16 points
Final score - a score agreed between the two reviewers

Supplementary Table 2b The results of quality evaluation and risk of bias assessment performed with JBI tool for qualitative studies

No.	Publication	1. Is there congruity between the stated philosophical perspective and the research methodology?	2. Is there congruity between the research methodology and the research question or objectives?	3. Is there congruity between the research methodology and the methods used to collect data?	4. Is there congruity between the research methodology and the representation and analysis of data?	5. Is there congruity between the research methodology and the interpretation of results?	6. Is there a statement locating the researcher culturally or theoretically?	7. Is the influence of the researcher on the research, and vice-versa, addressed?	8. Are participants, and their voices, adequately represented?	9. Is the research ethical according to current criteria or, for recent studies, and is there evidence of ethical approval by an appropriate body?	10.Do the conclusions drawn in the research report flow from the analysis, or interpretation, of the data?	No. of 'YES'	No. of 'YES'*2 + No. of 'UN'*1	Overall quality score	Overall risk of bias
		Final Score	Final Score	Final Score	Final Score	Final Score	Final Score	Final Score	Final Score	Final Score	Final Score			% of maximum score (in case of 'NA' the maximum score was adjusted)	LOW ≥ 70%; MODERATE = 50-69%; HIGH ≤ 49%
1	Bisconsin-Júnior [2022]	Yes	Yes	Yes	Yes	Yes	No	No	Yes	Yes	Yes	7	14	70	LOW
2	Bogueva [2022]	Yes	Yes	Yes	Yes	Yes	No	No	Yes	Yes	Yes	7	14	70	LOW
3	Cai [2021]	Yes	Yes	Yes	Yes	Yes	No	No	Yes	Yes	Yes	7	14	70	LOW
4	Grasso [2020]	Yes	Yes	Yes	Yes	Yes	No	No	NA	NA	Yes	5	10	38	HIGH
5	Gravely [2018]	Yes	Yes	Yes	Yes	Yes	No	Yes	Yes	No	Yes	7	14	70	LOW
6	Pérez-Lloréns [2020]	NA	NA	NA	NA	NA	NA	NA	NA	No	Yes	1	2	50	MODERATE
7	Reverberi [2021]	No	No	Yes	UN	UN	No	No	Yes	No	UN	2	7	35	HIGH

Note:
UN - unclear
NA - not applicable

Yes - 2 points
Unclear - 1 point
No - 0 points
In case of NA - the maximum score was adjusted respectively
Maximum score (when all criteria were rated 'Yes') = 20 points
Final score - a score agreed between the two reviewers
